# Dual Molecular Catalyst-Based Tandem That Enables Electrocatalytic CO_2_−Formaldehyde−Methanol Cascade Conversion

**DOI:** 10.1021/jacs.5c00316

**Published:** 2025-06-03

**Authors:** Arnab Ghatak, G. Shiva Shanker, Yanai Pearlmutter, Adi Fryder, Ran Shimoni, Idan Hod

**Affiliations:** Department of Chemistry and Ilse Katz Institute for Nanoscale Science and Technology, 26732 Ben-Gurion University of the Negev, Beer-Sheva 8410501, Israel

## Abstract

Electrocatalytic CO_2_ reduction into multielectron products is a promising approach for carbon capture and utilization. Recently, cobalt phthalocyanine (CoPc)-based molecular catalysts have shown potential competence toward electrochemical conversion of CO_2_ to methanol, a 6e^−^/6H^+^ product. Yet, despite the recent advancements, CoPc’s tendency to aggregate and the weak CO-intermediate binding generally limit its electrocatalytic activity and selectivity. Herein, we demonstrate that a metal−organic framework (MOF) could be used to construct a tandem electrocatalytic system via immobilization of 2 types of molecular catalysts (CoPc and Fe-porphyrin). Notably, the MOF-based tandem achieves a 3-fold increase in electrocatalytic CO_2_-to-methanol activity and selectivity compared to a CoPc-only MOF-based catalyst (up to 18% methanol faradaic efficiency at 25 mA/cm^2^). Additionally, operando spectroscopy and electrochemical analysis show that unlike typical tandem systems, the MOF-based tandem operates uniquely by using a reactive intermediate different from CO (i.e., formaldehyde). Hence, this proof-of-concept approach offers a new means to design molecular electrocatalytic schemes capable of driving complex proton-coupled electron transfer reactions.

## Graphical abstract

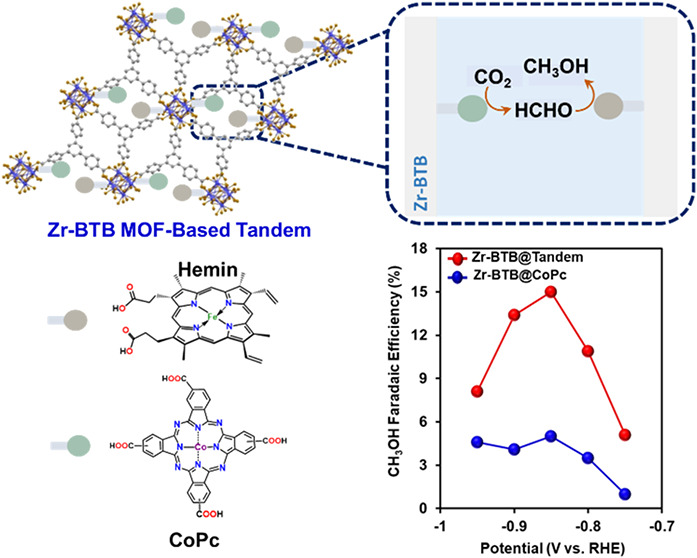

## Introduction

Fossil fuel combustion continues to increase the atmospheric CO_2_ levels, making it one of the major sources of greenhouse effect.^1^ Hence, there is an urgent need to develop new approaches to CO_2_ utilization. Among those, the electroreduction of CO_2_ to value-added C*_n_* chemicals has taken the center stage for carbon recycling and energy storage.^2–4^ Nevertheless, challenges in gaining fast reaction rates and appropriate product selectivity requisite the design of suitable electrocatalysts.^5,6^ In that respect, molecular electrocatalysts based on first-row transition elements have well-defined active sites and accurately tailorable structures for mechanism-based performance.^7–9^ Yet, typically they tend to follow the 2e^−^/2H^+^ CO_2_ reduction reaction (CO_2_RR) pathways, e.g., generating carbon monoxide (CO), formic acid (HCOOH), or oxalate (C_2_O_4_^2−^), owing to the complex intermediate species and reaction mechanisms involving the generation of multielectron products.^10–18^

Recently, several research efforts have been focused on using molecular catalysts to electrochemically reduce CO_2_ to multielectron products.^8,19,20,14^ Specifically, electrocatalytic CO_2_ reduction into a 6e^−^/6H^+^ product, methanol (CH_3_OH), is of particular interest due to its uses as a chemical building block for a wide variety of industrial chemical commodities.^21^ Ever since the pioneering work by Kapusta and Hackerman,^22^ examples of molecular catalysts that are active toward electroreduction of CO_2_ to CH_3_OH are mainly restricted to systems based on cobalt phthalocyanine (CoPc), operating via a sequential CO_2_−CO−CH_3_OH conversion mechanism.^23,24^ Yet, due to CoPc’s tendency to aggregate and its weak CO binding strength (compared to CO_2_ binding), these catalysts generally suffer from limited CH_3_OH selectivity. Recently, to overcome these limitations, CoPc was mounted over multiwalled carbon nanotubes (MWCNTs), thus achieving catalyst monodispersion and stabilizing the CO-bound intermediate by inducing bending strains on the deposited catalyst.^7,25–27^

In this context, metal−organic frameworks (MOFs) have demonstrated the capabilities to function as a heterogeneous, porous platform for electrochemical activation of small molecules via (1) immobilization of a large concentration of electrolyte-accessible molecular catalysts^28–35^ (while providing mass-transport conduits accessible for diffusion of ions and catalytic substrates toward the active sites) and (2) modulating the immediate chemical environment of the active site to tune catalysis.^36–43^ Despite that, so far there has been no attempt to exploit the unique chemical modularity of MOFs to assemble an electrocatalytic tandem system, composed of more than one type of tethered molecular catalyst and separated from one another by molecular length scales (i.e., few nm’s).^44^

Hence, in the present work, we have postulated that by constructing a MOF-based tandem system, through the installation of both CoPc and Fe-porphyrin (Hemin), one could generate efficient electrochemical CO_2_-To-CH_3_OH conversion. Our initial notion was that this catalytic scheme will (i) induce spatial separation between CoPc moieties, thus avoiding their aggregation and (ii) use Hemin to increase the CO_2_-To-CO generation, thus alleviating the CO/CO_2_ binding competition by increasing the local CO concentration in the vicinity of CoPc.

To do so, we covalently attached both CoPc and Hemin on the Zr_6_-oxo node of a 2D-Zr-BTB MOF and used the Zr-BTB@Tandem system as an efficient heterogeneous CO_2_RR electrocatalyst (Figure 1). The cooperative behavior of the two active sites significantly improved the 6e^−^/6H^+^ reduction of CO_2_ to CH_3_OH, achieving a maximum faradaic efficiency (FE) of 18% and a partial current of 24 mA/cm^2^ (at −1 V vs RHE) in a gas diffusion electrode (GDE) setup. However, despite our initial expectations that CoPc-bound CO will act as the tandem-influenced intermediate, operando Fourier transform infrared (FTIR) spectroscopy and electrochemical analysis revealed that the catalytic tandem reaction proceeds via electroreduction of the CoPc-bound formyl (−CHO) intermediate, while the role of the hemin catalyst is to drive the reaction from the formyl stage to CH_3_OH formation (Figure 1). For direct CO_2_RR, to the best of our knowledge, this constitutes the first reported electrocatalytic tandem system that utilizes a reaction intermediate other than CO to improve catalytic activity and selectivity (Wang et al. have shown that co-reduction of CO_2_ with nitrate generates a C−N coupling product, methylamine, on CoPc via a bound HCHO intermediate).^45^ Hence, these results should provide insights into the future design of more complex electrocatalytic tandem architectures.

**Figure 1:**
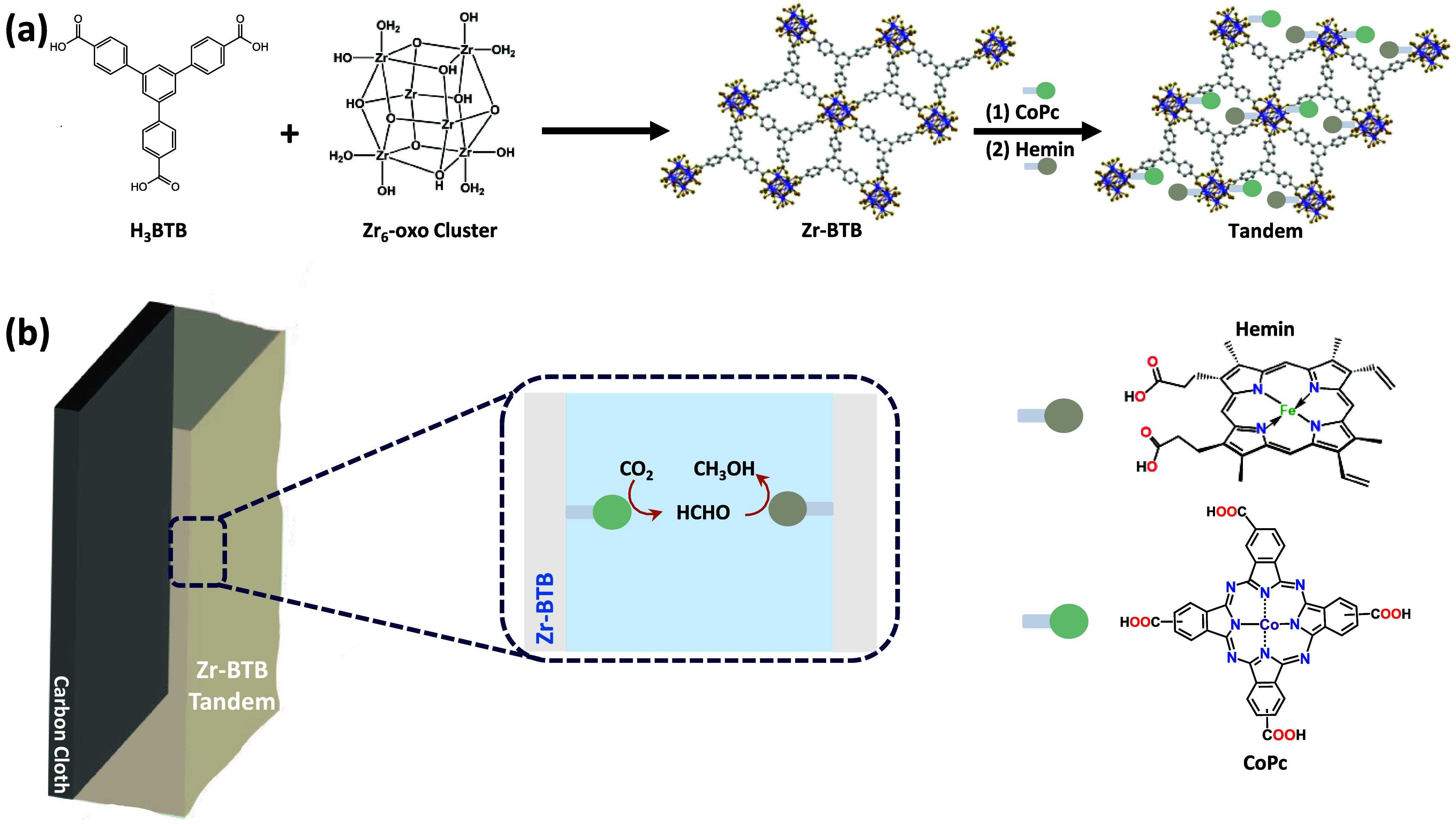
(a) Illustration of Zr-BTB MOF’s structure and its subsequent postsynthetic modification with CoPc and hemin to construct the tandem catalyst. (b) Schematic representation of the tandem’s sequential electrochemical CO_2_-To-CH_3_OH conversion principles, indicating the specific role of each catalyst.

## Results and Discussion

In order to prepare the aforementioned tandem catalyst system for CO_2_ electroreduction to a multielectron/multiproton product, we chose to employ a Zr_6_-oxo-based two-dimensional metal–organic framework (2D-MOF), Zr-BTB, as the platform due to its chemical robustness and favorable mass-transport properties. 2D layered Zr-BTB MOF was grown according to a previous literature report.^39^ Thereafter, the as-synthesized MOF was postsynthetically modified with CoPc, Hemin, or both catalysts via the solvent-assisted ligand incorporation (SALI) method, thus generating three catalyst systems: Zr-BTB@CoPc, Zr-BTB@Hemin, and Zr-BTB@Tandem (Zr-BTB@CoPc + Zr-BTB@Hemin), respectively (see Supporting Information for detailed experimental procedures).

First, the different MOF materials were characterized to verify their successful fabrication. Scanning electron microscopy (SEM) images of Zr-BTB MOF showed the formation of a typical 2D flower-like layered nanosheet morphology (Figure S1a). Further SEM analysis confirmed that the MOF’s morphology was maintained throughout the synthesis of the other three postmodified systems, i.e., Zr-BTB@CoPc (Figure S1b), Zr-BTB@Hemin (Figure S1c), and Zr-BTB@Tandem (Figure S1d). In addition, powder X-ray diffraction (PXRD) data collected for all samples indicated that the crystal structure of the parent 2D-MOF (Zr-BTB) was retained throughout the postsynthetic modifications (Figure S1e). Energy-dispersive X-ray spectroscopy (EDS) mapping and line-scan characterization revealed a homogeneous incorporation of Co in Zr-BTB@CoPc, Fe in Zr-BTB@Hemin, and both Co and Fe within the tandem catalyst, respectively (Figures S2–S4). Inductively coupled plasma optical emission spectrometry (ICP-OES) analysis was performed to quantitatively measure the catalyst surface loadings for all samples, indicating a molar ratio of 1.72 CoPc per Zr_6_-oxo MOF node for Zr-BTB@CoPc, 1.83 hemin per node Zr-BTB@Hemin, and a CoPc/hemin molar ratio of 1.15 for Zr-BTB@Tandem (Table S1). Finally, X-ray photoelectron spectroscopy (XPS) measurements were performed to further characterize the postsynthetically modified MOF systems (see Figure S5 and relevant discussion in SI).

Then, to perform electrochemical characterization of the different systems, electrodes of each MOF sample were prepared by drop-casting 135 μL of an ink solution (containing 30 mg of the MOF sample in a 2 mL solution of 75% water and 25% 2-propanol and 140 μL of Nafion binder) over carbon cloth conductive substrates, which ultimately resulted in 2 mg/cm^2^ of MOF surface loading. Electrochemical analysis was carried out in a gastight H-cell three-electrode setup in 0.5 M KHCO_3_ aqueous electrolyte solution. Ag/AgCl (in saturated KCl), Pt plate, and MOF-coated carbon cloths served as reference, counter, and working electrodes, respectively. (The electrode potential scale was converted to RHE during data representation.) The working and reference electrodes were separated from the counter electrode by an ion-exchange membrane, Nafion 117.

As seen in Figure S6a,b, to examine the electroactivity of the different MOF catalysts, cyclic voltammetry (CV) analysis (conducted in Ar-saturated 0.5 M KHCO_3_ aqueous electrolyte) of Zr-BTB@CoPc and Zr-BTB@Hemin was done, which revealed well-resolved quasi-reversible redox peaks corresponding to CoPc-based Co^II/I^ and Co^I/0^, having an *E*_1/2_ of 132 and −402 mV, respectively, and Hemin-based Fe^III/II^ and Fe^II/I^, with an *E*_1/2_ of 153 mV and −370 mV, respectively. Similarly, the CV of the Zr-BTB@Tandem system showed two peaks, combining the overlapping redox features attributed to both CoPc and Hemin (Figure S6c). For all samples, plotting of CoPc’s and Hemin’s redox peaks current as a function of the square root of the CV scan rate showed a linear dependence, thus confirming a diffusion-controlled charge transport process (Figure S6d–f), as expected in redox-active MOFs, in which charge propagation is governed by an electron-hopping conduction mechanism.^31,38^

To characterize the electrocatalytic CO_2_RR performance of the different samples, a comparison of linear sweep voltammetry (LSV) data in Ar-saturated and CO_2_-saturated 0.5 M KHCO_3_ electrolyte solution was performed, showing a catalytic current corresponding to CO_2_RR by all the three MOF systems (Figure S7a–c). Notably, although the current density for the catalytic CO_2_RR is almost identical for Zr-BTB@Tandem (Figure S7d, red trace) and Zr-BTB@CoPc (Figure S7d, blue trace), a slightly higher current density was obtained with the tandem system at the highest overpotential, while the lowest current density was recorded for Zr-BTB@Hemin (Figure S7b). Moreover, the potential of catalytic onset (*E*_onset_) also varied with the catalyst systems, having the lowest value for the tandem at −0.35 V, followed by −0.47 V for Zr-BTB@CoPc and −0.52 V for Zr-BTB@Hemin (Figure S7d). Note that *E*_onset_ has been determined by drawing tangents in the non-Faradaic zone (conventionally, horizontal lines) and the Faradaic zone of the LSV curve, while the abscissa of the point of intersection of these tangents gives the onset potential value.

Next, the electrocatalytic CO_2_RR activity of all the three catalytic systems was measured via bulk-electrolysis experiments conducted in the potential range of −0.75 to −0.95 V vs RHE, passing a constant charge of 20C per potential (Figures S8–S10). Catalytic product analysis at each potential was conducted to determine the gaseous products: CO and H_2_ (quantitatively measured using gas chromatography (GC) analysis of the reaction’s headspace, Figure S11), and the liquid product: CH_3_OH (quantitatively measured using ^1^H NMR spectra of the post-electrolysis solution, Figure S12). For experiments done under ^12^CO_2_ atmosphere, ^1^H NMR spectra of the post-electrolysis solution showed a characteristic ^12^CH_3_OH single peak at ∼3.25 ppm (Figure S12). When we repeated the experiment in ^13^CO_2_-saturated KH^13^CO_3_ electrolyte solution, the singlet peak in the ^1^H NMR spectrum splitted into a doublet at 3.09 and 3.39 ppm (Figure S12), thus indicating the formation of ^13^CH_3_OH and proving that the source of methanol is CO_2_RR.

Figure 2a–c plots the combined FE of CH_3_OH (blue), CO (gray), and H_2_(red) as a function of the applied potential for all three catalysts. (Calculation of FE for all CO_2_RR products is provided in SI.) For both Zr-BTB@CoPc and the Zr-BTB@Tandem systems, the total FEs (for CH_3_OH (FE_CH_3_OH_) + CO (FE_CO_) + H_2_ (FE_H_2__)) were calculated to be higher than 90% (at potentials between −0.8 and −0.9 V vs RHE). Notably, the Zr-BTB@Tandem catalyst produced CH_3_OH with increased selectivity from −0.75 V until reaching a maximum FE of 15% at −0.85 V vs RHE (Figure 2d, red trace) corresponding to a turnover frequency (TOF) of 0.43 s^−1^. Table S2 shows the comparison of the catalytic activity of molecular catalysts for CO_2_ to CH_3_OH formation in terms of TOF. Zr-BTB@CoPc also followed the same trend for the FE of CH_3_OH, albeit obtaining a maximum FE_CH_3_OH_ of only 5% (at −0.85 V, Figure 2d, blue trace). In the case of the other two major products, CO and H_2_, the trend showed the FE for CO to be much higher in the case of Zr-BTB@CoPc (maximum of 60% at −0.85 V, Figures 2b andS13a, blue trace) compared to the tandem (maximum of 36% at −0.85 V, Figures 2a and S13a, red trace), while the H_2_ production efficiency followed the opposite trend (Figure S13c). In addition, to understand the manner in which the tandem system affects the rate of CH_3_OH production, the partial current density of methanol (*j*_CH_3_OH_) was plotted as a function of the applied potentials (Figure 2e). Interestingly, for all applied potentials, one can observe that the catalytic rate of CH_3_OH generation is substantially elevated for the dual molecular catalyst-based MOF-tandem compared to the CoPc-modified MOF, Zr-BTB@CoPc. Specifically, Zr-BTB@Tandem enhanced the CO_2_-To-CH_3_OH kinetics by up to 374% at −0.9 V vs RHE. As a result, it is evident that compared to the single-catalyst system, the CoPc and hemin-based tandem system significantly improved both the CO_2_RR activity and selectivity. Yet, the tandem’s operation mechanism is still unknown. In this respect, the exact role of the Zr-BTB MOF platform is crucial for sustaining catalytic operation, as the screening with bare CoPc catalyst shows substantially diminished activity (Figure S13), due to the lowered fraction of electroactive species because of catalyst aggregation. A discussion regarding the role of the MOF in our system is provided in SI, following Figure S13.

**Figure 2:**
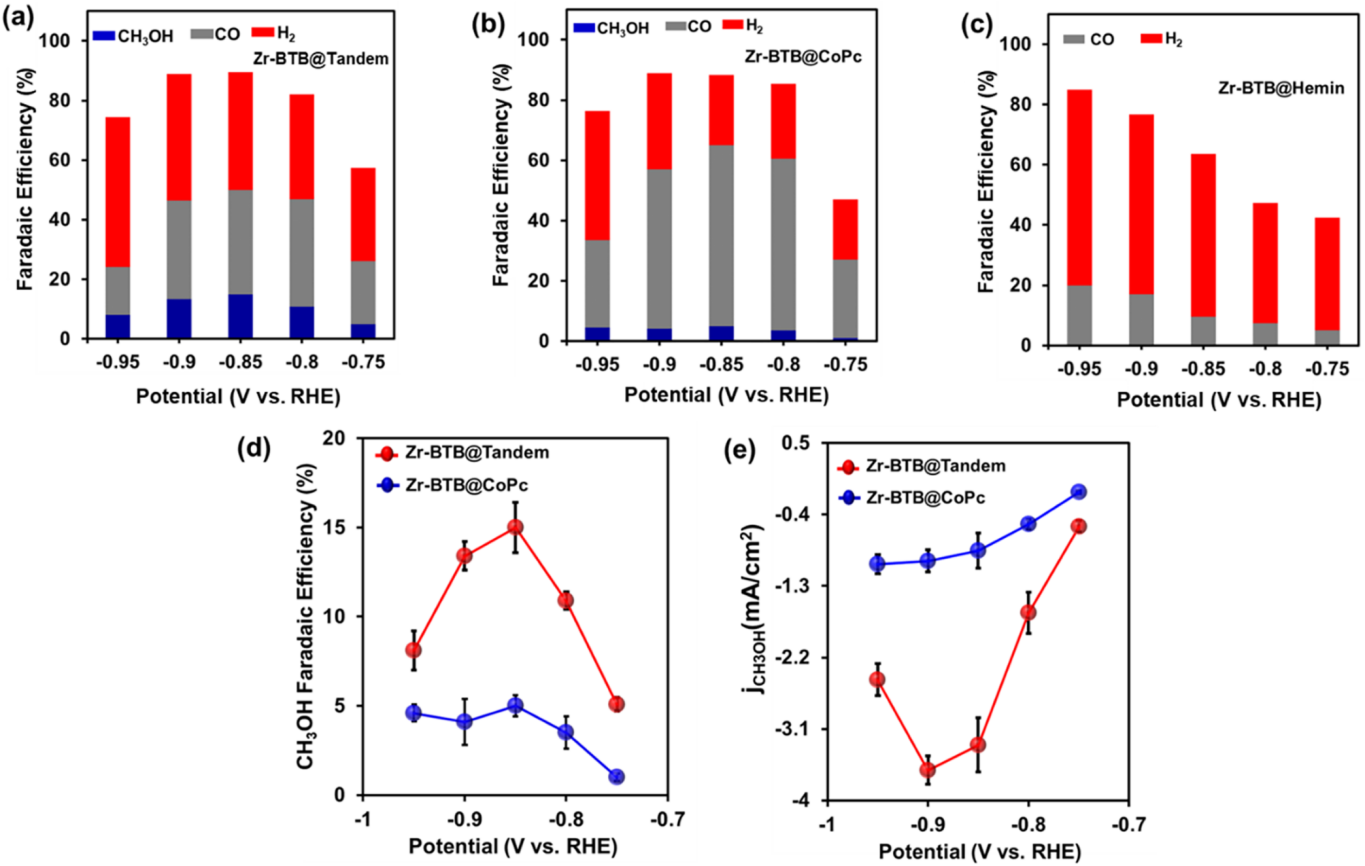
Bar diagram indicating the selectivity of CH_3_OH, CO, and H_2_ for the three catalyst systems: (a) Zr-BTB@Tandem, (b) Zr-BTB@CoPc, and (c) Zr-BTB@Hemin. (d) CO_2_RR Faradaic efficiency of CH_3_OH formation vs potential for Zr-BTB@Tandem (red trace) and Zr-BTB@CoPc (blue trace) in the potential range of −0.75 to −0.95 V vs RHE. (e) CO_2_RR partial current density (*j*_CH_3_OH_) vs potential for Zr-BTB@Tandem (red) and Zr-BTB@CoPc (blue).

Additionally, we investigated the manner in which MOF-installed CoPc-to-Hemin molar ratios affect the tandem’s reactivity and selectivity toward methanol production. To this end, we prepared a series of Zr-BTB@Tandem by varying the equivalence of CoPc and hemin inside the MOF (Table S3). As we systematically increased the CoPc-to-hemin molar ratio from 0.38 to 1.15, the selectivity of FE increased from 4.8% to the maximum value of 15% (at an applied potential of −0.85 V vs RHE). Further increasing the CoPc-to-Hemin molar ratio, viz. 4.80, decreased the FE of MeOH to 12% at −0.85 V (Table S3). This indicates that an optimum concentration of both CoPc and Hemin is required for the formation of the tandem catalyst in order to obtain maximum output.

As mentioned earlier, our initial assumption was that hemin would efficiently convert CO_2_ to CO, increase its local availability next to the CoPc catalyst, and thus facilitate CH_3_OH generation. However, as clearly seen in Figures 2c and S14a,b (brown trace), at applied potentials relevant to the Zr-BTB@Tandem’s operation, the Zr-BTB@Hemin system produced CO at a very low selectivity and rate. Instead, the FE of H_2_ is high, systematically increasing from 37% at −0.75 V to 65% at −0.95 V vs RHE (Figure S13c). In other words, Hemin’s operation principles within the tandem system must be different from what was expected.

Consequently, to get insight into the mechanism of the tandem electrocatalytic CO_2_RR system, we employed operando infrared reflection absorption spectroscopy in ATR (ATR-IRRAS) under Otto configuration (see SI for experimental information). To collect these spectro-electrochemical measurements, Zr-BTB@CoPc and Zr-BTB@Tandem catalysts were drop-casted over glassy-carbon (GC) electrodes, and in-operando FTIR data were collected in a CO_2_-saturated 0.5 M KHCO_3_ aqueous electrolyte solution, in the CO_2_RR-relevant potential range (−0.75 to −0.95 V), and compared against background data collected at 0 V vs RHE, where CO_2_RR does not take place. As can be seen in Figure 3a, in the case of the Zr-BTB@Tandem system, as the applied potential was swept cathodically, a gradual rise of a band at 1654 wavenumber was observed, which was absent in the 0 V data. We note that the band is located just beside the bending-mode band of water. Hence, to further investigate the origin of this vibration, isotope labeling experiments were performed both in ^13^CO_2_-saturated 0.5 M KH^13^CO_3_ solution in H_2_O and in ^12^CO_2_-saturated 0.5 M KHCO_3_ solution in D_2_O. In the ^13^CO_2_-saturated electrolyte solution, the 1654 cm^−1^ band shifted to 1610 cm^−1^ (Figure 3b), while in D_2_O solution containing ^12^CO_2_ the 1654 cm^−1^ band shifted to 1630 cm^−1^ (Figure S15a). Thus, the 1654 cm^−1^ band, along with its 44 cm^−1 12/13^C shift and 24 cm^−1^ H/D secondary shift, matches well with those of the previously reported CoPc-bound formyl (−CHO) intermediate species.^46–48^

**Figure 3:**
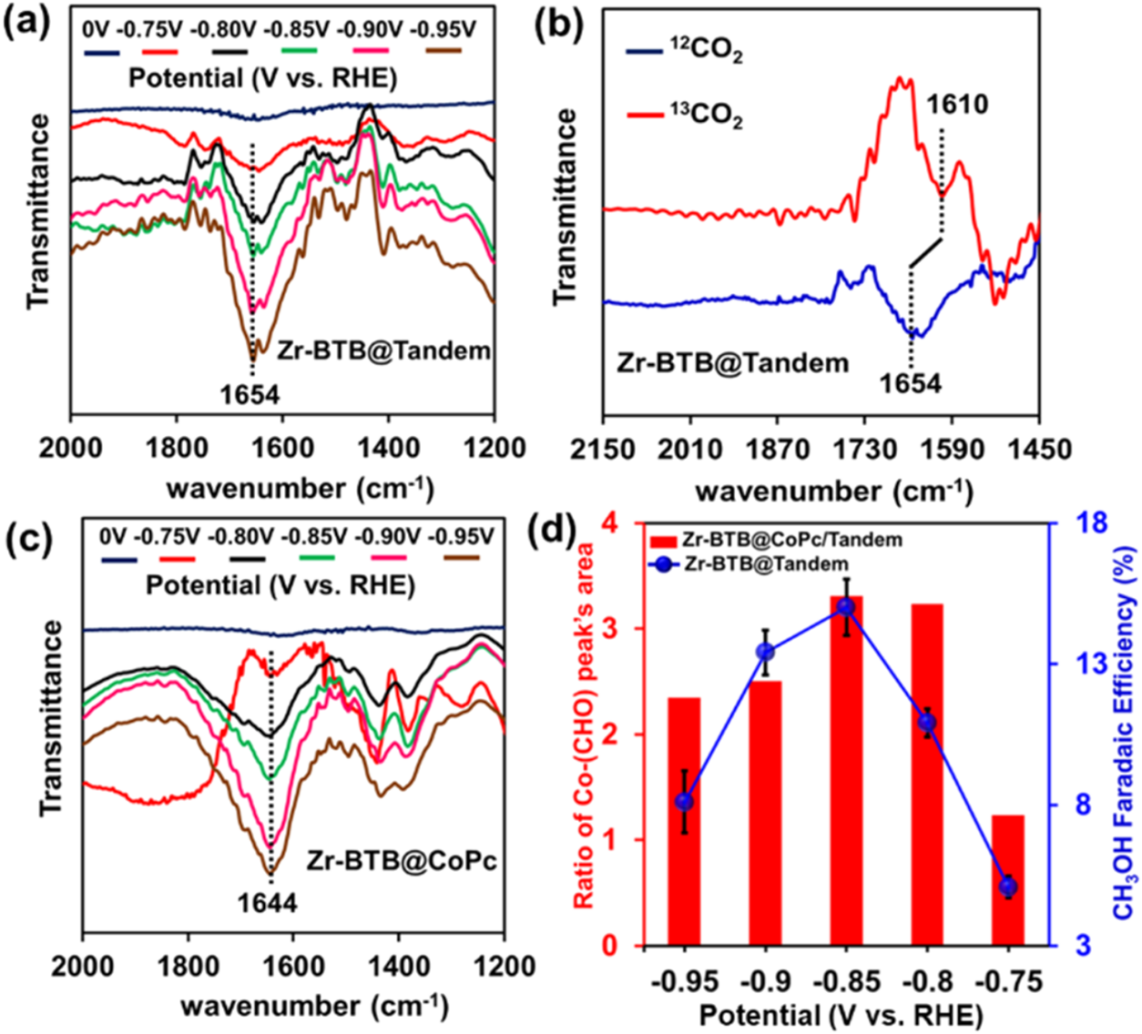
(a) In-operando ATR-IRRAS spectra of Zr-BTB@Tandem catalyst in ^12^CO_2_-saturated 0.5 M aqueous KHCO_3_ electrolyte starting from −0.75 V vs RHE (red trace) to −0.95 V vs RHE (brown trace). The spectrum taken at 0 V vs RHE (blue trace) was considered as a background, where CO_2_RR does not take place. (b) Overlay of the ATR-IRRAS spectrum of the Zr-BTB@Tandem catalyst at −0.95 V vs RHE in ^12^CO_2_-saturated KH^12^CO_3_ solution (blue trace) and in ^13^CO_2_-saturated KH^13^CO_3_ solution (red trace). (c) In-operando ATR-IRRAS spectra of the Zr-BTB@CoPc catalyst starting from −0.75 V vs RHE (red trace) to −0.95 V vs RHE (brown trace). The spectrum taken at 0 V vs RHE (blue trace) was considered as a background, where CO_2_RR does not take place. (d) Plot comparing the ratio of Co-(CHO) peak’s area (Zr-BTB@CoPc/Zr-BTB@Tandem red bar) and the Zr-BTB@Tandem’s CO_2_-To-CH_3_OH selectivity (blue trace), as a function of the applied potential.

The operando FTIR data for Zr-BTB@CoPc indicated a similar rise of a CoPc-bound formyl band at increasing cathodic potentials, located at 1644 cm^−1^ (Figure 3c). Importantly, we observed that for all applied potentials, the relative intensity of Zr-BTB@CoPc’s Co-bound −CHO peak was considerably higher than that of the Zr-BTB@Tandem catalyst. This implies the fact that the tandem system facilitates the consumption of the formaldehyde intermediate during catalysis. Moreover, Figure 3d plots the ratio of the −CHO peak area of Zr-BTB@CoPc/Zr-BTB@Tandem (red bars) alongside Zr-BTB@Tandem’s FE_CH_3_OH_ (blue trace) as a function of the applied potential. One can see that both −CHO peak’s area ratio and Zr-BTB@Tandem’s FE_CH_3_OH_ potential dependence go hand in hand, jointly reaching a maximum value at potential of −0.85 V. These results signal a tight correlation between the tandem’s ability to consume the formaldehyde intermediate and its overall elevated efficiency toward CO_2_-To-CH_3_OH catalytic conversion.

Further evidence for the fact that the tandem system exhibits improved electrocatalytic methanol production activity is found through a scrutiny of Zr-BTB@Tandem’s operando FTIR data at increasing cathodic potentials in the high-frequency region, showing the rise of two bands located at 2844 and 2915 cm^−1^ (which were absent in the Zr-BTB@CoPc data), previously assigned to the C−H stretching vibrations *v*(C−H) of the metal-bound methoxy (−OCH_3_) species (Figure S15b).^49,50^ On top of that, the Zr-BTB@Tandem’s low-frequency-region infrared (IR) data showed the corresponding C−H bending of the metal-bound OCH_3_, i.e., δ­(C−H) species, at 1346 cm^−1^ (which was also absent in the case of Zr-BTB@CoPc) (Figure S15c).^16^ For Zr-BTB@Hemin, the metal-bound formyl species were not detected (Figure S15d and the overlay data in S15e). Thus, essentially, the in-operando spectro-electrochemical data suggests that the role of hemin in the tandem system is to promote the 2e^−^/2H^+^ reduction of formaldehyde, forming a metal-bound methoxy intermediate, which in turn increases the rate and selectivity of CH_3_OH formation for the tandem system compared to Zr-BTB@CoPc.

As such, to validate our notion regarding the tandem’s CO_2_RR operation mechanism, we performed electrolysis experiments using formaldehyde (HCHO) as the catalytic substrate (instead of CO_2_). First, LSV data were collected for all three catalysts, in an electrolyte solution containing depolymerized paraformaldehyde (30 mM) in 0.5 M aqueous KHCO_3_ (under an Ar environment). As seen in Figure 4a, Zr-BTB@Hemin’s onset potential of electrocatalytic HCHO reduction (−0.68 V vs RHE) was lower compared to the one of Zr-BTB@Tandem and Zr-BTB@CoPc (−0.78 V vs RHE), thus hinting at Hemin’s HCHO reduction capability. Additionally, for each catalyst, chronoamperometric electrolysis experiments were done at CO_2_RR relevant potentials and the corresponding ^1^H NMR analysis indicated the formation of MeOH as the HCHO reduction product. Importantly, Zr-BTB@Hemin exhibited the highest selectivity toward HCHO reduction to CH_3_OH. Specifically, at the potential of −0.85 V (potential of peak catalytic CO_2_RR performance), FE_CH_3_OH_ was calculated to be 10.4,18, and 25% for Zr-BTB@CoPc, Zr-BTB@Tandem, and Zr-BTB@Hemin, respectively (Figure 4b). Furthermore, the rate of HCHO-To-CH_3_OH conversion was also monitored by plotting the CH_3_OH partial current densities, *j*_CH_3_OH_. As shown in Figure 4c, Zr-BTB@Hemin’s HCHO-To-CH_3_OH rate is considerably faster than that of Zr-BTB@CoPc. Nevertheless, the Zr-BTB@Tandem system’s catalytic rate is the highest out of the 3 catalysts, since its total current density is the largest (Figure 4a red curve) and much higher compared to Zr-BTB@Hemin. Thus, electrocatalytic HCHO reduction experiments reinforce the mechanistic pathway of reduction of CO_2_ to CH_3_OH, as suggested by the operando FTIR data. The role of hemin in the tandem catalysis is mainly to drive the reaction from the aldehyde intermediate stage toward CH_3_OH evolution.^47,51^ In the absence of hemin (Zr-BTB@CoPc), a higher population of Co-bound −CHO intermediate gets accumulated over the electrode during the CO_2_RR.^47^ Note that since Zr-BTB@Hemin itself is not capable of driving the CO_2_RR to formaldehyde, it cannot promote an overall CO_2_ conversion to CH_3_OH, thus only generating H_2_ and CO as catalytic products.

**Figure 4:**
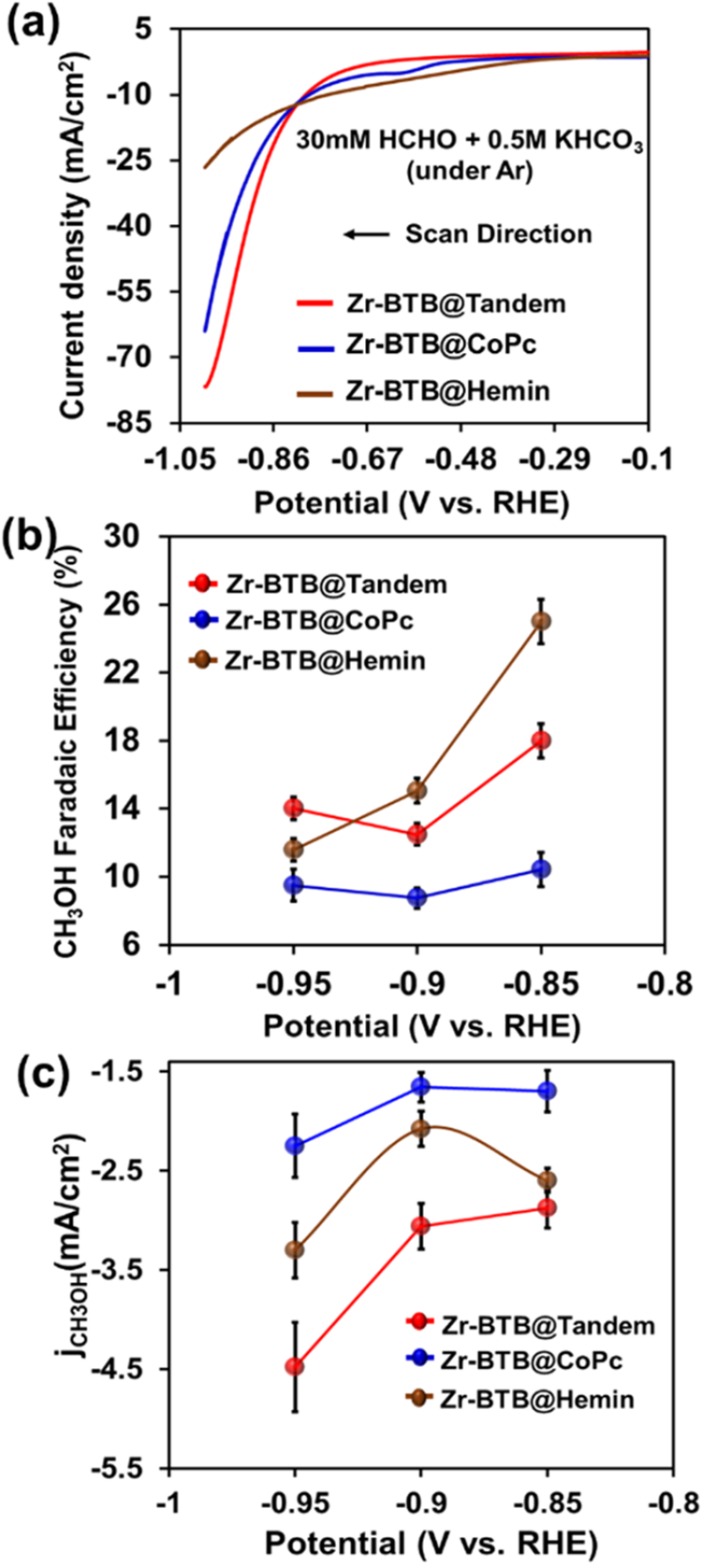
(a) LSV data for HCHO (30 mM) reduction in 0.5 M KHCO_3_ comparing the Zr-BTB@Tandem catalyst (red trace), Zr-BTB@CoPc (blue trace), and Zr-BTB@Hemin (brown trace). (b) Faradaic efficiency of CH_3_OH formation in HCHO reduction vs potential for Zr-BTB@Tandem (red trace), Zr-BTB@CoPc (blue trace), and Zr-BTB@Hemin (brown trace) in the potential range of −0.85 to −0.95 V. (c) Partial current density (*j*_CH_3_OH_) vs potential (V vs RHE) for Zr-BTB@Tandem (red), Zr-BTB@CoPc (blue), and Zr-BTB@Hemin (brown) in case of HCHO reduction.

Finally, we were set to examine the CO_2_RR performance of our 2D-MOF-based tandem catalyst in a more practical gas diffusion electrode (GDE) flow-cell electrochemical setup. To do so, the Zr-BTB@Tandem catalyst was drop-casted on a 20% PTFE-coated gas diffusion layer (GDL) using an ink prepared with poly­(vinylidene fluoride) (PVDF) binder using *N*-methyl-2-pyrrolidone (NMF) solvent. All flow-cell electrochemical measurements (linear sweep voltammetry/chronoamperometry) were done in recirculation mode, where the electrolyte was recirculated throughout the experiment, while the CO_2_ gas flow was allowed a single pass (see further experimental details in SI). As shown in Figure 5a, LSV of the Tandem GDL electrode under electrochemical CO_2_RR conditions revealed a catalytic onset potential of −0.70 V vs RHE. Markedly, the observed catalytic current densities were ∼5 times higher than the ones recorded using the conventional H-cell (in a potential window of −0.75 to −1.05 V vs RHE). Chronoamperometric bulk-electrolysis measurement (at potential of −1.0 V) showed a similar CH_3_OH selectivity as with the H-cell configuration. Specifically, it exhibited an initial FE_CH_3_OH_ of 18% (after 30 min of electrolysis), and this selectivity was largely maintained throughout the 4 h of electrocatalytic operation (Figure 5b). Moreover, the rate of CH_3_OH production in GDE was substantially accelerated compared to the conventional H-cell. As seen in Figure 5c, the GDE’s *j*_CH_3_OH_ remained essentially constant throughout the electrolysis, with a maximum partial current of-25.4 mA/cm^2^, ∼6 times larger than that of the H-cell. (Table S4 in the SI summarizes all error bars and average values for all major data sets discussed in this paper.)

**Figure 5:**
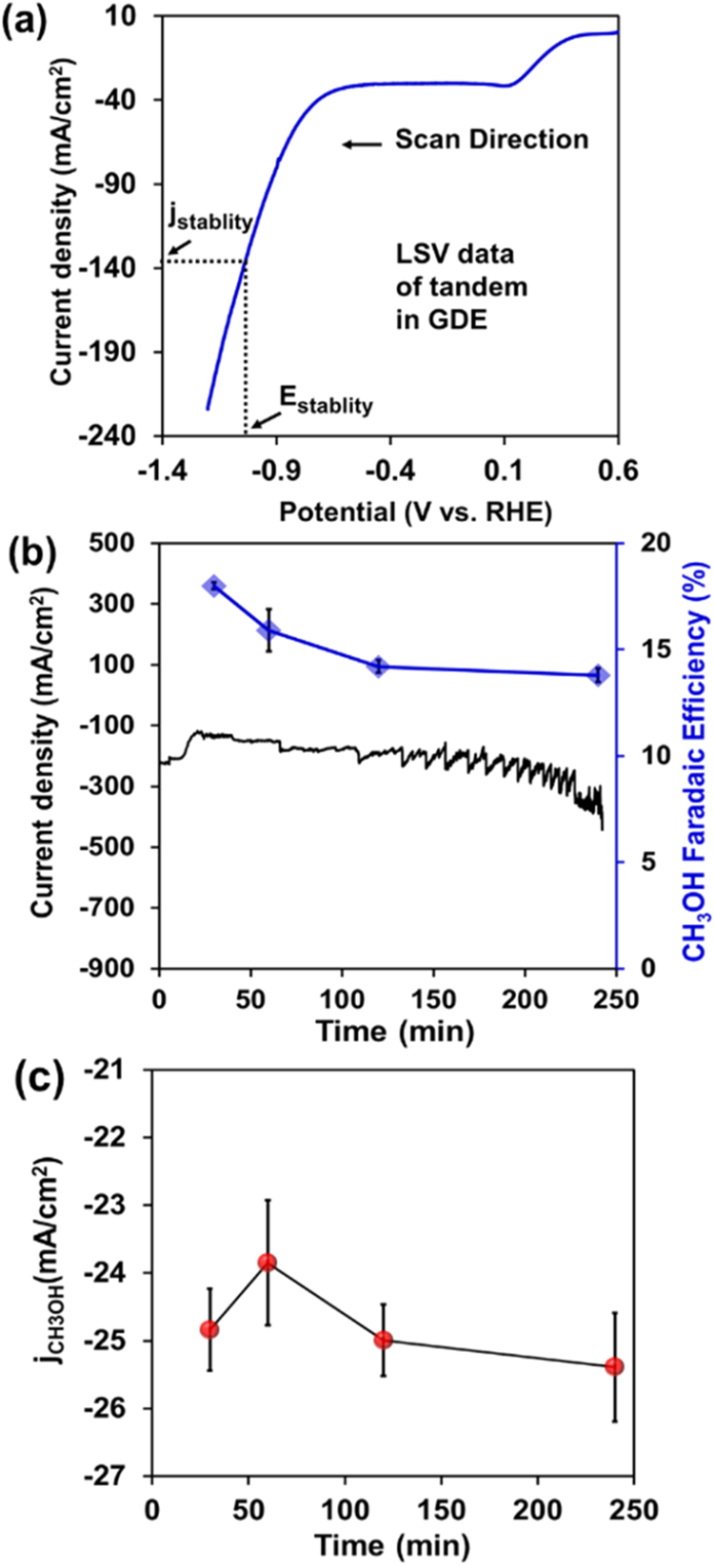
(a) LSV data of CO_2_RR by the Zr-BTB@Tandem catalyst system in GDE, indicating the potential and current density at which the stability test is done. (b) Current density plot as a function of time (mins.) for the Zr-BTB@Tandem catalyst system at −1.0 V vs RHE for eCO_2_RR in GDE (black trace), with the corresponding selectivity of %FE CH_3_OH shown by the blue trace for 250 min time. (c) Partial current density of methanol (*j*_CH_3_OH_) as a function of time (mins.) for Zr-BTB@Tandem in case of CO_2_RR in GDE.

We further tried to detect and quantify HCHO as an intermediate during the CO_2_RR in GDE by our systems. As shown in Figure S16, formation of a trace amount of HCHO as an intermediate was detected for Zr-BTB@CoPc, whereas Zr-BTB@Tandem does not produce HCHO at all as an intermediate from the CO_2_RR. This is in strong agreement with the suggested mechanism and role of hemin in the MOF-based tandem system.

Lastly, upon 4 h of electrolysis, post-catalytic stability analysis of the GDL electrode was performed via XRD and SEM (Figure S17). The structure of the Zr-BTB@Tandem catalyst was found to be unaltered by the bulk electrolysis as the morphology was retained. Thus, the tandem catalyst system can be considered a durable one for synthesis of MeOH from CO_2_ in an aqueous medium. A comparative metric discussion regarding the efficacy of the Zr-BTB@Tandem system in GDE with respect to other contemporary reports is shown in SI.

To conclude, we have demonstrated that a MOF could be used as a platform to assemble a dual molecular catalyst-based tandem scheme, capable of efficient electrocatalytic CO_2_ reduction to a 6e^−^/6H^+^ product, CH_3_OH. Specifically, immobilization of both CoPc and Hemin within a 2D-Zr-BTB MOF triples the rate and selectivity of CO_2_-To-CH_3_OH conversion (maximum FE_CH_3_OH_ of 15% at *j*_CH_3_OH_ of 3.62 mA/cm^2^). Additionally, performing the CO_2_RR with a GDE setup enabled a 6-times higher conversion rate with similar selectivity (18%) as that of an H-cell configuration. Typically, CO_2_RR tandem electrocatalysts utilize CO as a median reactant to generate multielectron products. Yet, in-operando FTIR and electrochemical characterization indicated that the MOF-based Zr-BTB@Tandem operates through a unique CO_2_−formaldehyde-CH_3_OH cascade mechanistic pathway. First, CoPc reduces CO_2_ to form an aldehyde intermediate species, while the role of hemin is to promote further reduction of the aldehyde to form CH_3_OH via a metal-bound methoxy intermediate. Hence, this work adds new molecular insights into the operation principles of electrocatalytic MOFs and should allow future design of catalytic schemes that drive complex proton-coupled electron transfer reactions.

## Supplementary Material



## Notes

Chemicals; experimental procedures; material synthesis and characterization; physical characterization methods and details of electrochemical experiments (PDF)
