# Supplementary material for: Dual Molecular Catalyst-Based Tandem That Enables Electrocatalytic CO2−Formaldehyde−Methanol Cascade Conversion
Source: J Am Chem Soc. 2025 Jun 3;147(24):20329–37. doi: 10.1021/jacs.5c00316 (PMC12186479; doi:10.1021/jacs.5c00316)
Supplement: Supplementary file 1 [file ja5c00316_si_001.pdf]

Supporting Information

Dual Molecular Catalyst-Based Tandem That Enables Electrocatalytic CO_2-Formaldehyde-Methanol Cascade Conversion

Arnab Ghatak,‡ G. Shiva Shanker,‡ Yanai Pearlmutter, Adi Fryder, Ran Shimoni and Idan Hod*

Department of Chemistry and Ilse Katz Institute for Nanoscale Science and Technology, Ben-Gurion University of the Negev, Beer-Sheva, 8410501, Israel.
E-mail: hodi@bgu.ac.il

Table of Contents

Section	Page Number
Table of Contents	**S2**
Synthetic Procedure	**S2-S3**
Ink Preparation	**S3**
Physical Characterisation Methods	**S3-S4**
Experimental Details and Sample Preparation	**S4-S5**
Supporting Figures and Relevant Discussion	**S7-S17**
References	**S17**

Synthetic Procedures

Chemicals

Zirconium (IV) chloride (ZrCl4 ≥ 99.5%), 1,3,5-tris(4-carboxyphenyl) benzene-H_3BTB ($C_{27}H_{18}O_6$ ≥ 98%), 4-Hydroxy benzoic acid ($C_7H_6O_3$ ≥ 98 %), Nafion perfluorinated solution (5 wt %), potassium bicarbonate ($KHCO_3$ ≥ 99 %), paraformaldehyde (powder ≥ 95%) were purchased from sigma aldrich. Hemin ($C_{34}H_{32}ClFeN_4O_4$, ≥ 98%) was purchased from Carl Roth. Cobalt (II) 2,9,16,23-tetra(carboxyl) phthalocyanine (CoPc, $C_{36}H_{16}CoN_4O_4$ ≥ 95%) was purchased from Porphychem.

Dimethylformamide (DMF, C_3H_7NO), Methanol (CH_4O) were purchased from Bio-Lab. Ethanol (C_2H_6O, 99.7%), 2-Propanol, (C_3H_8O, 99%) were purchased from J.T Baker. Carbon cloths (thickness:0.33mm, basic weight 120 g/m2, resistance < 5mΩcm2) were provided by Gochen ltd. CO_2 and Ar gas (≥ 98%) were purchased from Maxima. SGL 28 BC with Graphene −20% FEPD 121 Filler-Gas diffusion layers (GDL) were purchased from FuelCellStore, and $^{13}CO_2$ gas (99% C, < 2% 18 O) was purchased from Cambridge isotope laboratory. For sonication, ultrasonic bath sonicator has been used with 37 kHz frequency.

Synthesis Procedures of

a) Zr-BTB

This synthesis has been done exactly following the previous literature report.[1] Briefly, $ZrCl_4$ (200 mg), H_3BTB (200 mg), benzoic acid (12 g), 10 mL water and 60 mL DMF were mixed in a 100 mL cupped bottle. The mixture was sonicated until dissolved and then heated at 120°c for 48 hr in an oven. After 48 hr the cupped bottle was cooled down to room temperature and the product was collected under centrifugation after washing several times with DMF and acetone. The product was dried in vacuum for overnight, to obtain layered flake like 2D-nanosheets of Zr-BTB MOF.

b) Zr-BTB@CoPc

60 mg of CoPc was mixed with 100 mg of Zr-BTB nanosheet in 10 mL DMF and sonicated until soluble (for 20 mins). The mixture was then heated to 70°c for 24 hr in an oven. After cooling down to room temperature the product was isolated via centrifugation after washing three times with DMF and three times with ethanol, until the solution of the supernatant liquid becomes colourless. The product was then vacuum dried for overnight, to obtain greenish black powder of Zr-BTB@CoPc.

c) Zr-BTB@Hemin

This is synthesised exactly following the previous literature report. Briefly, 80 mg of Hemin was mixed with 100 mg of Zr-BTB nanosheet in 10 mL DMF and sonicated for 15 mins. The solution was then heated to 70°c for 24 hr in an oven. After cooling down to room temperature the product was isolated via centrifugation after washing three times with DMF and three times with ethanol, until the solution of the supernatant liquid becomes colourless. The product was then vacuum dried for overnight, to obtain black powder of Zr-BTB@Hemin.

d) Zr-BTB@Tandem

25 mg of Zr-BTB@CoPc and 18mg of Zr-BTB@Hemin were weighed in 20mL vial, and mixed in 15mL DMF. The mixture was then sonicated for an hour. Post that, the solution was heated in 70°c for 24 hr in an oven. After cooling down to room temperature, the product was isolated via centrifugation after washing with toluene (twice) and ethanol (twice). Finally, the mixture was vacuum dried for overnight, to obtain Zr-BTB@Tandem catalyst.

Ink Preparation

15 mg of each catalyst was mixed in 1 mL of 75% water, 25% isopropanol and to the mixture 70 μL of Nafion 117 binder was added. The mixture was sonicated until a homogeneous solution was obtained. A carbon cloth working electrode (width 1 cm and length 2 cm) was coated with 135 μL of the ink, so that the surface coverage becomes 2 mg/cm^2(dry weight).

Physical Characterization Methods

As synthesised Zr-BTB, Zr-BTB@CoPc, Zr-BTB@Hemin and Zr-BTB@Tandem crystalline structure were confirmed using X-ray diffraction of PANalytical's Empyrean multi-purpose diffractometer instrument and Cu-Kα (0.15405 nm) radiation.

Scanning electron microscope (SEM) images were recorded using Verious XHR 460L SEM instrument operating at 2kV accelerating voltage. Images were recorded at different magnifications. Energy Dispersive X-ray Spectroscopy (EDS) analysis was performed to estimate the concentration ratio between Zirconium, Cobalt and Iron. EDS data and line scanning was done using the software OXFORD INSTRUMENTS.

The (XPS) spectra were collected using ESCALAB 250 apparatus X-ray photoelectron spectrometer, with Al-Ka X-ray source and monochromator. The survey spectra were recorded with a pass energy (PE) 150 eV and the high energy resolution was achieved with PE of 20 eV. To correct the charging effect, all measured spectra were calibrated relatively to carbon 1s peak position at 284.8 eV and the data processing was done with AVANTGE program.

Experimental Details and Sample Preparation

Electrochemical Measurements

A two-compartment gas tight H-cell using a three-electrode set up was used for the electrochemical measurements. One compartment contained the working and reference electrode while the counter electrode was placed in the other compartment. The two compartments were separated by a Nafion 117 membrane. Unless mentioned otherwise, catalyst drop casted carbon cloth electrode (active area 1 cm^2) was used as working electrode. Ag/AgCl in sat. KCl and Pt foil (with an active area of 1 cm^2) were used as reference and counter electrodes, respectively. All electrochemical experiments were performed at ambient temperature with Bio-Logic VSP instrument. All the experiments were performed using 0.5 M aqueous $KHCO_3$ electrolyte solution. Before starting each experiment, the

electrolyte solution was purged with Ar for 30 mins and in case of catalysis experiment this is followed by purging with CO_2 gas for another 30 mins to make the pH of the solution 6.8. All electrochemical experiments were performed at ambient temperature, and electrode potentials were converted to the RHE scale using the relation E(RHE)=[E(Ag+/Ag)–(0.059×pH)+0.197V]. iR correction was done before each experiment. The linear sweep voltammograms during catalysis were collected at 50 mV/sec scan rate, and other scan rates for cyclic voltammetry experiments were mentioned in the respective plots. Every polarization curve that we report in the manuscript is only with the steady state current density, which is obtained after the first cycle and remains stable. For product analysis chronoamperometric measurements were performed at -0.75V to -0.95V vs. RHE with 100 mV intervals and 20C charge were passed in each experiment.

Flow Cell Measurements

The electrochemical experiments were performed using a homemade plexiglass flow cell that consists of three compartments: an anolyte, catholyte, and gas-fed chambers. Nickel foam, catalyst-coated gas diffusion electrode (GDE), and Ag/AgCl (saturated KCl) were used as anode, cathode, and reference electrode, respectively. In all the experiments, the geometric area of the catalyst coating on the GDE was maintained 1.5 cm x 1.5 cm at the cathode for the CO_2 reduction reaction. The loading of the catalyst was maintained of 1.25 mg/cm^2 area. 1.0 M $KHCO_3$ electrolyte solution was used in both the anolyte and the catholyte chambers, separated by a proton exchange membrane (Nafion 117). The electrolytes were made using 18.2 MΩ·cm milli-Q water. The electrolyte in the reservoir was first purged with Argon to remove the oxygen and then saturated with CO_2 gas (usually purged 40 min) before conducting the electrochemical experiments. The pH (7.4) of the electrolyte was measured after purging with CO_2 for 40 min.

All flow-cell electrochemical measurements (linear sweep voltammetry/chronoamperometry) were done by recirculation mode, the electrolyte was recirculated throughout the experiment, and CO_2 gas was allowed a single pass. During the electrochemical measurements, the anolyte and catholyte chambers were pumped by an electrolyte solution at a volumetric flow rate of 20 mL/min through the recirculation mode from the separate reservoirs containing 40 mL of 1.0 M $KHCO_3$ electrolyte solution using a digital pump (Reodox.me). The gas feed CO_2 was supplied from the back side of the GDE electrode at a flow rate of 30 mL/min in single-pass mode, and the flow rate was measured by a mass-flow controller (Alicat Scientific). Likewise, the outlet gas flow rate was also measured using a mass-flow controller. The linear sweep voltammograms (LSVs) were taken at 50 mV/sec scan rate in 1.0 M $KHCO_3$ electrolyte solution for CO_2 electroreduction reaction. The 50C charge was passed through the working electrode using chronoamperometry measurement at each potential. All electrochemical experimental data was collected by applying IR compensation (85%) to correct the voltage loss.

After the electrolysis, the electrolyte solution was extracted from the cathodic compartment connected reservoir to determine the faradic efficiency of the products. The extracted electrolyte solution was analyzed using a ^{1}H NMR (500 MHz) spectrometer using a water (H_2O) suppression program. The NMR sample was prepared by mixing 0.45 mL electrolyte and 0.1 mL D_2O solution, and a 0.05 mL known concentration of dimethylsulfoxide (DMSO) solution was used as an internal standard. The concentration of methanol was calculated by comparing the peak area of methanol (3.28 ppm (chemical shift)) to that of the internal standard DMSO.

We conducted long-term stability experiments for Zr-BTB@Tandem coated GDE at -1.0 V potential vs. RHE for 4 hours and with specific time intervals aliquots were taken out from the electrolyte solution to check the selectivity. The chronoamperometric measurements were repeated multiple times using different batches of tandem catalyst-coated electrodes.

All electrode potentials were converted to the RHE scale by following the equation ($E_{RHE} = E_0 + 0.059 \times pH + 0.197$ V).

Preparation of Ink and Fabrication of Gas Diffusion Electrode (GDE)

20 mg of Zr-BTB@Tandem catalyst and 2.5 mg (12.5 wt%) polyvinylidene fluoride (PVDF) were taken in a 5 mL vial, and 1 mL of N-Methyl-2-pyrrolidone (NMF) solvent was added to the mixture. Then, the mixture was stirred on a magnetic stirrer for more than 12 hrs to form homogeneous ink. 1.25 mg/cm^2 of Zr-BTB@Tandem catalyst was coated on a 1.5 cm x 1.5 cm area on 20% PTFE (Polytetrafluoroethylene) coated GDE, then dried the electrodes in a programable hot oven at 60° C for 30 to 40 min. The uniform coating of catalyst on GDEs was used in all the electrochemical measurements.

Note: While drying electrodes in the oven, leaving them longer time than mentioned causes cracks on the electrode surface, which leads to inconsistency in the results.

Electrolyte of ^{13}CO2 saturated 0.5M $KH^{13}CO_3$ was prepared following the previous literature,[2] where $^{13}CO_2$ was bubbled through 0.5M KOH solution for 25 minutes. While doing the checking with $^{12}CO_2$, pH reached the neutral condition in a similar set up with KOH solution.

In case of formaldehyde electrochemical reduction experiment 30 mM initial concentration. of formaldehyde was used by dissolving in 0.5M $KHCO_3$ solution after pH adjustment. The calculation for the concentration of formaldehyde from the polymer paraformaldehyde was done using the calculation method shown by Boutin etal. in the previous literature reports.[2]

Formaldehyde Quantification Experiment

Formaldehyde was quantified using ^{1}H NMR by following the report of Mark Robert and co-workers.[3] In this method, formaldehyde reacts with sodium bisulfite ($NaHSO_3$) and forms the HCHO-sodium bisulfite adduct in the electrolyte solution. The reaction between HCHO and $NaHSO_3$ is thermodynamically favorable, and the resultant obtained product was stable for days.

First, we added a series of known concentrations of HCHO solution to the 1 M $NaHSO_3$ solution (50: 50 v/v) and stirred overnight using the magnetic stirrer to form the HCHO-sodium bisulfite adduct. The obtained adduct was taken to ^{1}H NMR (500 MHz), which gave a signal ~at 4.28 ppm indicating that the formation of the adduct was consistent with the prior report and from that a calibration curve was obtained.

To quantify formaldehyde in our electrolysis samples, 500 C charge was applied at -0.95 V vs. RHE through a working electrode using a chronoamperometric experiment for Zr-BTB@CoPc, and Zr-BTB@Tandem catalyst coated GDE catalysts using flow cell conditions (see an experimental section for details). After the electrolysis, the electrolyte solution was taken from the catholyte compartment separately from both the measurements and the same procedure was applied for the formation of the adduct mentioned above.

Experimental Details of ATR-IRRAS (Attenuated Total Reflectance-Infrared Reflection Absorption Spectroscopy in Otto Configuration)

We followed the similar procedure and setup for in-situ experiments with FTIR coupling with electrocatalysis, as discussed in our previous literature.[4, 5] For all the ATR-IRRAS measurements, 0.5 M $KHCO_3$ aqueous electrolyte solution purged with Ar (20 mins) initially, followed by 25 minutes purging

with $^{12}CO_2$ or $^{13}CO_2$ depending on the experiments flowed through the cell with a flow rate of 15 ml/min and then background was collected before starting the experiments. In case of in-situ data collection chronoamperometric measurements were performed for 5 minutes at each 50-mV interval within the potential range of (-0.75V vs RHE.) – (-0.95 V vs RHE.) and compared with the chronoamperometric measurement done at 0V vs. RHE.

Sample Preparation for ICP-OES Analysis

To determine the amount of Zr, Co and Fe in Zr-BTB@CoPc, Zr-BTB@Hemin, and Zr-BTB@Tandem respectively, ICP-OES analysis was performed using a Spectro ARCOS ICPOES, FHX22 multi-View plasma (SOP, EOP) instrument. To prepare the sample, each sample of measured amount (~ 2mg) was digested in 5 ml of conc. HNO_3 at 150 °C for 12 hours. 1 ml of the acid solution was diluted to 10 mL for ICP-OES measurement.

Sample Preparation for ^1H NMR Experiments

Hydrogen Nuclear Magnetic Resonance (^1H-NMR) measurements were done on the Bruker DPX-500 instrument. A calibration curve of CH_3OH was prepared using standard samples of known concentration in D_2O with DMSO as internal standard to evaluate the concentration of product formed during electrocatalysis. During the electrocatalysis experiment, post electrolysis solution (aliquot of 500 μL) was dissolved in D_2O with internal standard DMSO so as to make the volume of the NMR tube 600 μL. ^1H nuclear magnetic resonance data collection was done (Bruker 400 MHz, USA), under water suppression method. A blank experiment was also performed as a control using the same method before conducting each experiment, where CO_2 saturated solution was not used.

Calculation of Faradaic Efficiency of CH_3OH

Under aqueous electrolyte solution of CO_2 saturated 0.5 M $KHCO_3$ (pH = 6.8), CO_2 is reduced according to the following equation

$CO_2 + 6e^- + 6H^+ = CH_3OH + H_2O$

So, basically 6 electrons are required for the CH_3OH formation.

Now, the faradaic efficiency was estimated from the charge consumed for CH_3OH production and the total charge passed through the electrode, according to the following equation

Faradaic Efficiency (FE)= $(nF*C_{CH3OH}*V)/Q$,

Where, n= no. of electrons required for the reduction, which is 6, according to the above equation

F= Faraday const. i.e. 96500 C mol^{-1}, C_{CH3OH} is the measured ammonia concentration, V is the volume of electrolyte used, which is 35 mL for each experiment and Q is the charge passed from the electrode which is maintained as 20C throughout all the experiments.

Supporting Figures and Relevant Discussions

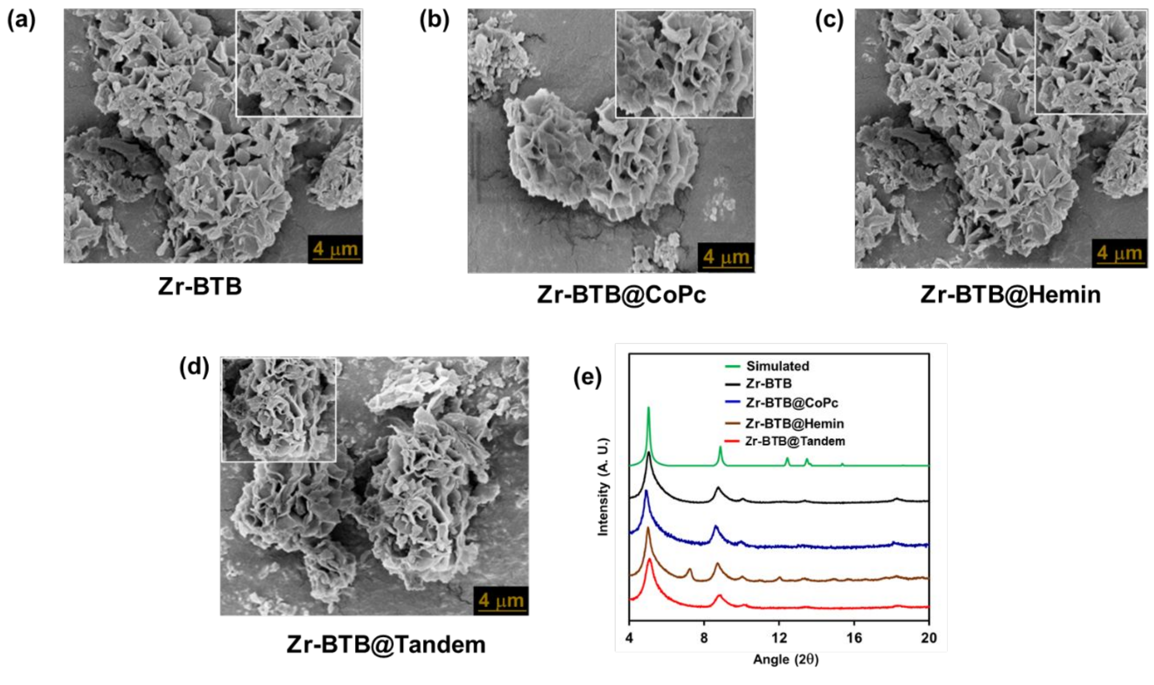

Figure S1. SEM images of (a) Zr-BTB, (b) Zr-BTB@CoPc, (c) Zr-BTB@Hemin and (d) Zr-BTB@Tandem catalysts respectively, and (e) PXRD pattern of simulated Zr-BTB, Zr-BTB, Zr-BTB@CoPc, Zr-BTB@Hemin- and Zr-BTB@Tandem respectively.

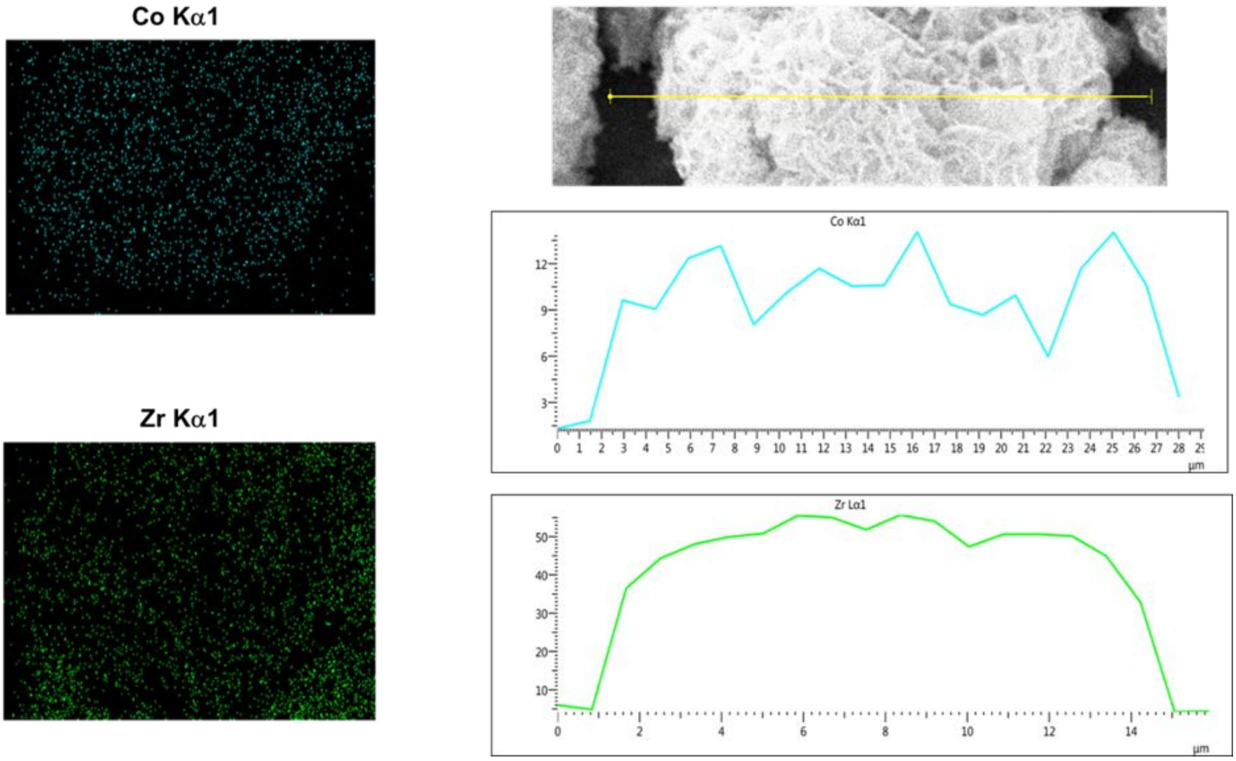

Figure S2. Elemental mapping with elemental line scanning from SEM analysis (Energy-dispersive X-ray spectroscopy (EDS)), for Zr-BTB@CoPc, showing the homogeneous dispersion of cobalt in the catalyst.

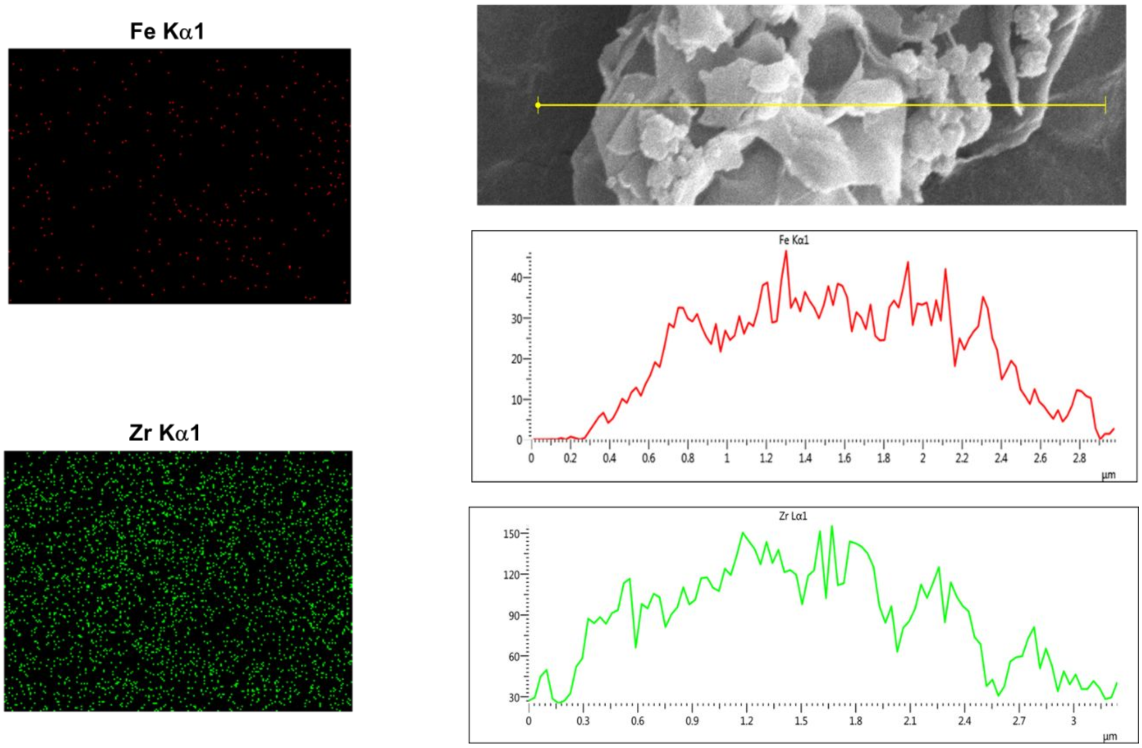

Figure S3. Elemental mapping with elemental line scanning from SEM analysis (Energy-dispersive X-ray spectroscopy (EDS)), for Zr-BTB@Hemin, showing the homogeneous dispersion of iron in the catalyst.

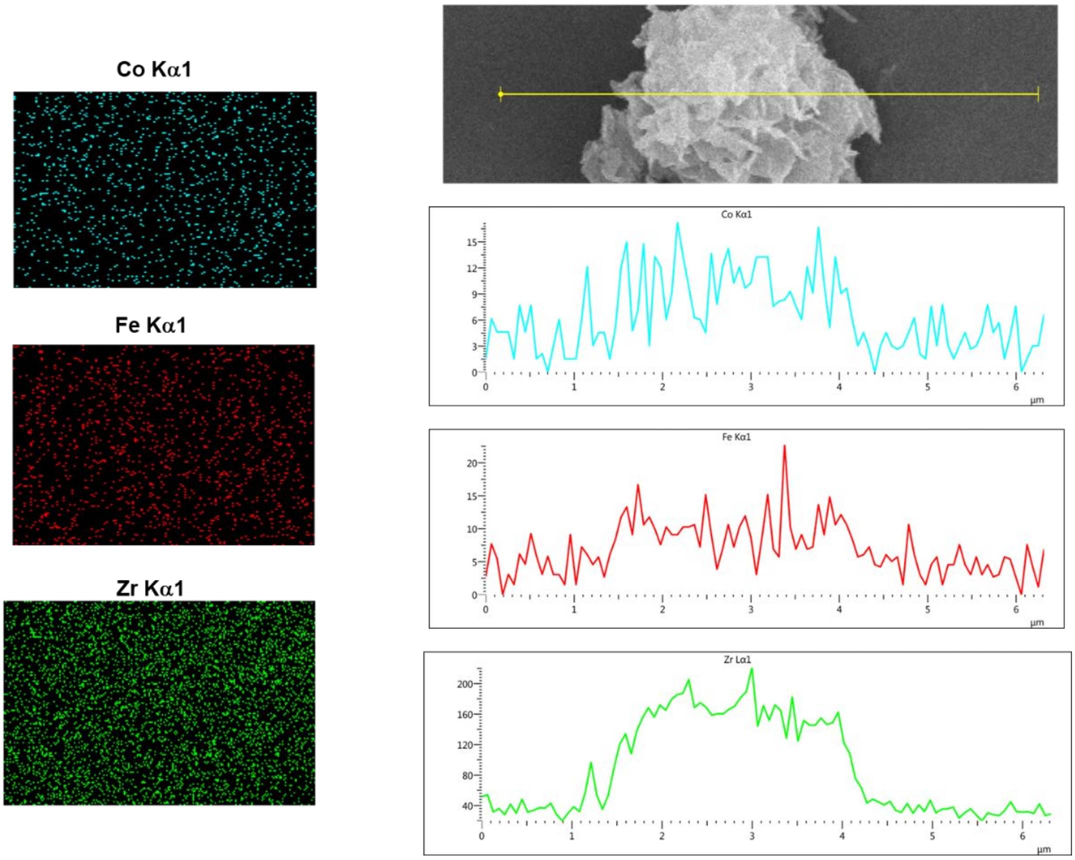

Figure S4. Elemental mapping with elemental line scanning from SEM analysis (Energy-dispersive X-ray spectroscopy (EDS)), for Zr-BTB@Tandem, showing the homogeneous dispersion of cobalt and iron both in the catalyst.

**Table S1.** Summary of ICP-OES analysis for all the catalysts										
Complex	ICP-OES Analysis									
	Zr (mg/L)	Fe (mg/L)	Co (mg/L)	Zr_6 µ mole	Fe µ mole	Co µ mole	Fe/Zr_6	Co/Zr_6	Co/Fe	CoPc+Hemin/Zr_6
Zr-BTB@CoPc	5.25	-	0.99	0.48	-	0.84	-	1.72	-	
Zr-BTB@Hemin	4.06	0.75	-	0.37	0.67	-	1.83	-	-	
Zr-BTB@Tandem	8.23	0.65	0.82	0.75	0.59	0.68	0.78	0.90	1.15	1.68

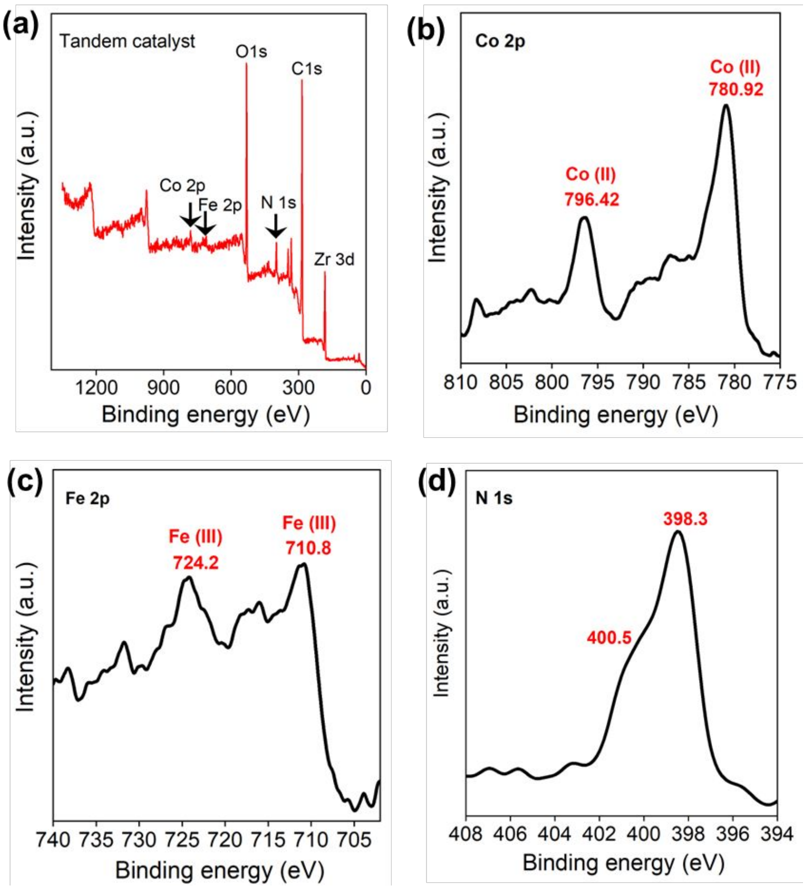

Figure S5. (a) XPS measurements showing the survey scans for Zr-BTB@Tandem catalyst. Corresponding Co2p, Fe2p and N1s analysis are shown in (b), (c) and (d) respectively. Due to catalyst formation the oxidation states of the atoms Co(II) and Fe(II) remains unchanged as has been shown from the data.

X-ray photo electron spectroscopy (XPS) measurements for the Zr-BTB@Tandem catalyst showed two major cobalt peaks (Figure S5b), one for Co $2p_{1/2}$ (796.42 eV) and another for Co $2p_{3/2}$ (780.92 eV) which fit well with the 2p subshell of Co in +II oxidation state,[6] present in the resting state of the catalyst. Similarly, (Figure S5c) showed the presence of iron in +III oxidation state in the resting form of the catalyst with Fe $2p_{1/2}$ peak at 724.20 eV and Fe $2p_{3/2}$ 710.80 eV,[4] indicating the successful incorporation of the cobalt and iron in the tandem system. N1s XPS data (Figure S5d) proved the incorporation of nitrogen as hemin and cobalt phthalocyanine inside the tandem,[4] and all of this didn't alter the properties of the MOF, indicated by the survey scan (Figure S5a).

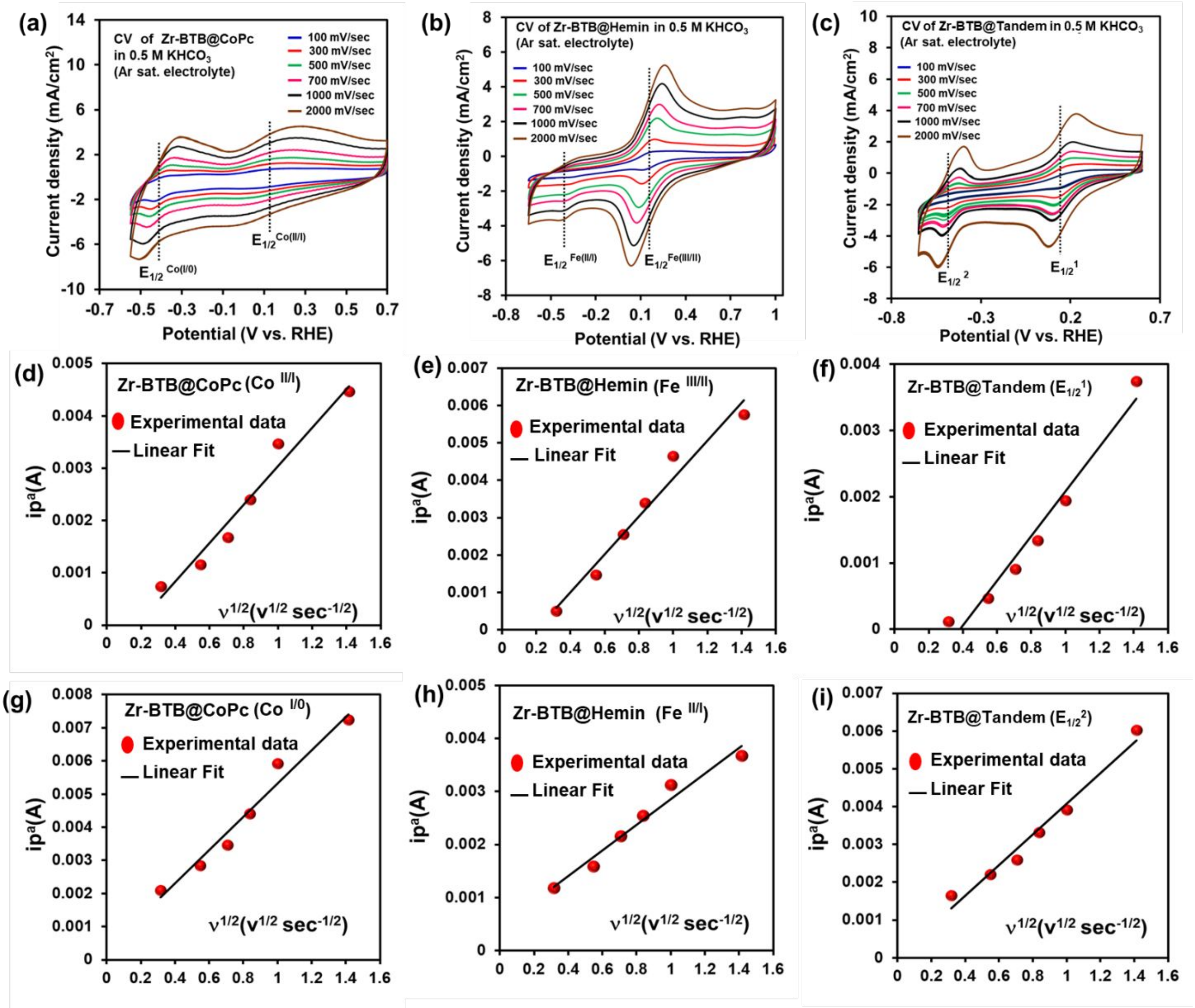

Figure S6. (a) CV data of Zr-BTB@CoPc in Ar saturated 0.5 M $KHCO_3$ solution at different scan rates from 100-2000 mV/sec, indicating two redox couples $Co^{II/I}$ ($E_{1/2}{}^1$) and $Co^{I/0}$ ($E_{1/2}{}^2$) respectively. (b) CV data of Zr-BTB@Hemin in Ar saturated 0.5 M $KHCO_3$ solution at different scan rates from 100-2000 mV/sec, indicating three redox couples $Fe^{III/II}$ ($E_{1/2}{}^1$), $Fe^{II/I}$ ($E_{1/2}{}^2$) respectively. (c) CV data of Zr-BTB@Tandem catalyst in Ar saturated 0.5 M $KHCO_3$ solution at different scan rates from 100-2000 mV/sec, indicating two redox couples combining that of CoPc and Hemin. From the CV data at different scan rates (ν), a plot of peak current (ip) vs. square root of scan rate ($\nu^{1/2}$) for all the three catalysts, (d) Zr-BTB@CoPc (Co $^{II/I}$) (g) Zr-BTB@CoPc (Co $^{I/0}$), and (e) Zr-BTB@Hemin (Fe $^{III/II}$) (h) Zr-BTB@Hemin (Fe $^{II/I}$), and (f) Zr-BTB@Tandem ($E_{1/2}{}^1$), (i) Zr-BTB@Tandem ($E_{1/2}{}^2$) respectively, has been done and it shows the linearity in almost all the cases, indicating that charge transfer process is diffusion controlled for all of them.

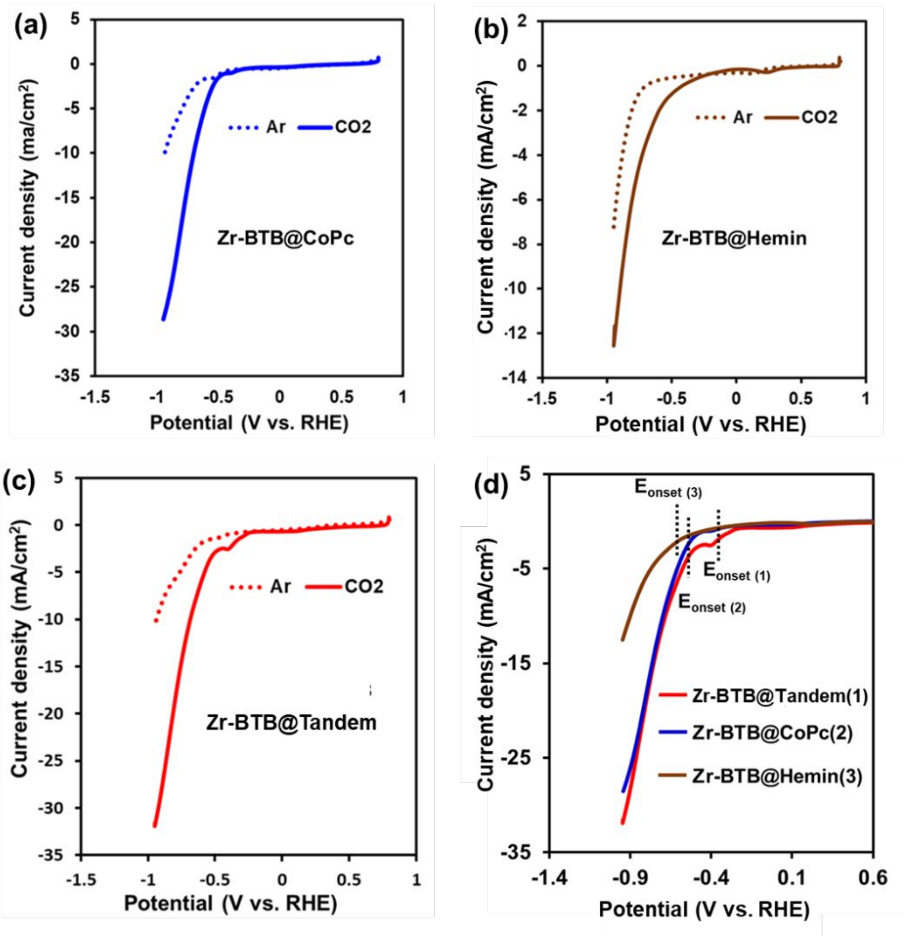

Figure S7. LSV data of the catalysts system under Ar atmosphere (dotted line) and under CO_2 atmosphere (solid line) in 0.5M $KHCO_3$ solution in the potential window of 1V to -1.5V vs. RHE for (a) Zr-BTB@CoPc, (b) Zr-BTB@Hemin and (c) Zr-BTB@Tandem catalysts respectively. (d) Overlay of LSV data under catalytic condition of CO_2-saturated 0.5M $KHCO_3$ solution for Zr-BTB@Tandem (red trace), Zr-BTB@CoPc (blue trace) and Zr-BTB@Hemin (brown trace) respectively, showing the onset potential with the dotted black for each catalyst system, where (1)- Zr-BTB@Tandem, (2)-Zr-BTB@CoPc and (3)-Zr-BTB@Hemin, respectively.

Onset potential in an electrochemical redox reaction (E_{onset}) is the low over potential where the Faradaic process becomes measurable or in other words Tafel plot is determined to deviate away from linearity i.e. the so-called Tafel region of a voltammogram where the concentration of the reagents at the interface are not significantly altered from that of the bulk solution. After that point in an electrocatalytic process, the current is expected to increase exponentially as a function of potential.[7] It has been determined by drawing tangents in non-Faradaic zone (conventionally horizontal line) and Faradic Zone of the LSV curve and the abscissa of the point of intersection of these tangents give the onset potential value.[8] In our manuscript, we determined the E_{onset} in each case by the same method.

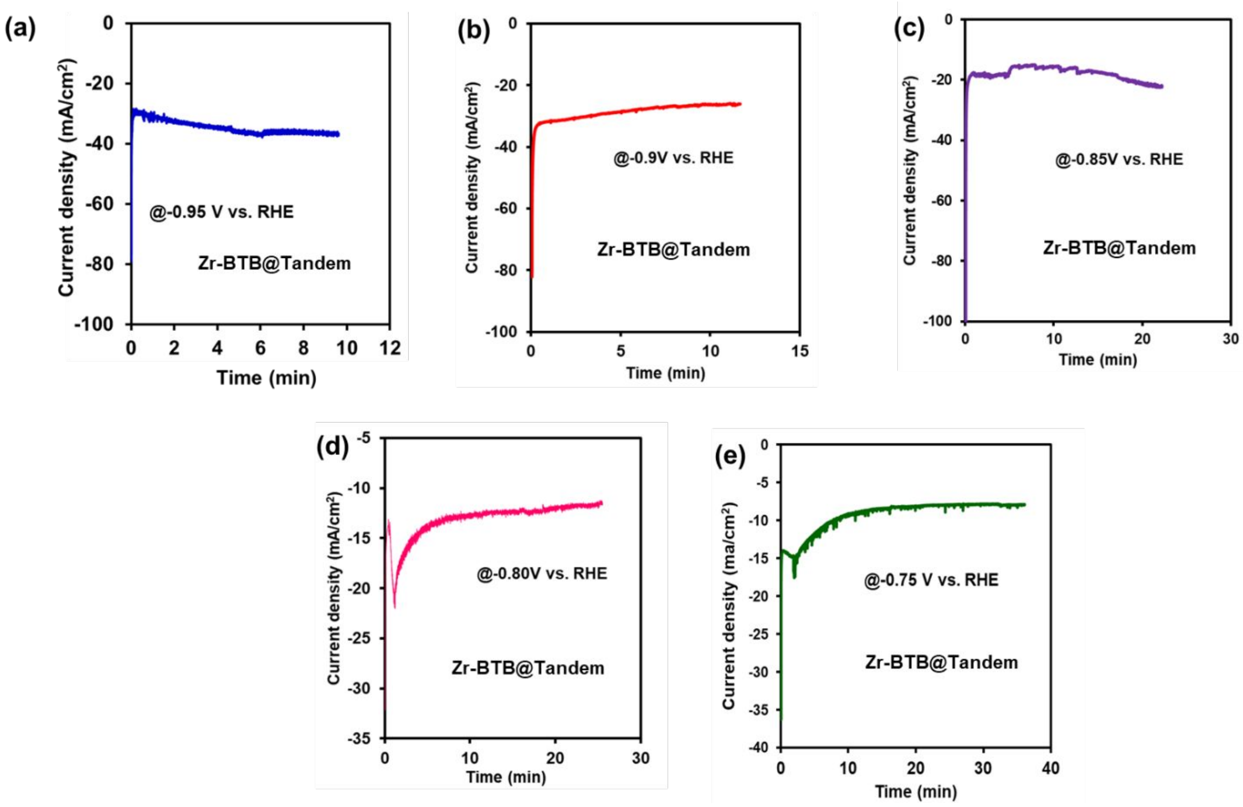

Figure S8. I vs. t (min) curves for the accumulation of 20C charge at all the potentials from -0.75V to -0.95V for Zr-BTB@Tandem catalyst in the chronoamperometric measurement.

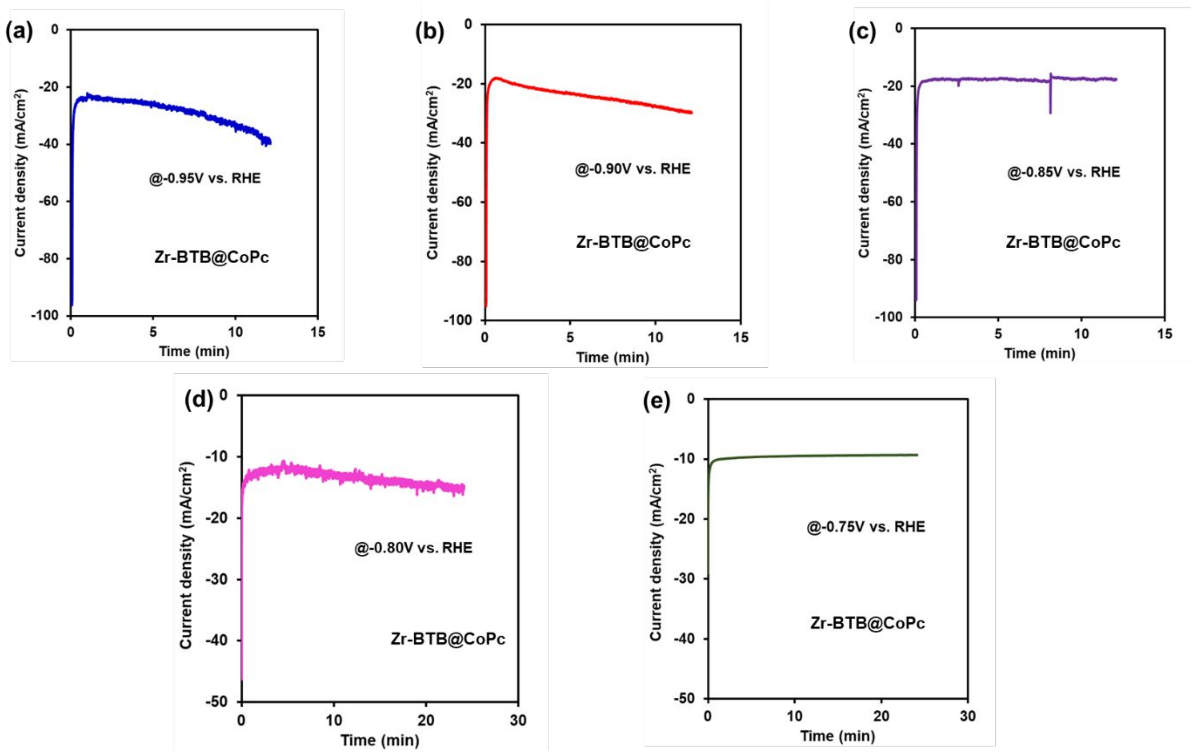

Figure S9. I vs. t (min) curves for the accumulation of 20C charge at all the potentials from -0.75 V to -0.95V for ZrBTB@CoPc in the chronoamperometric measurement.

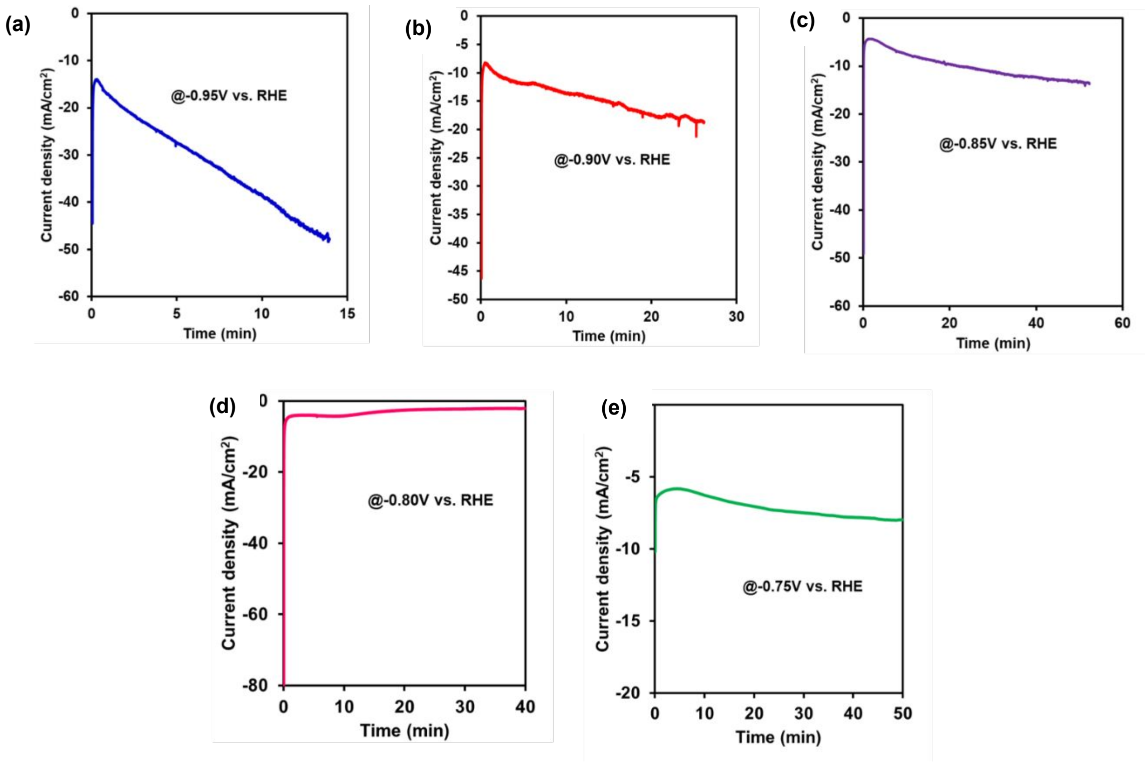

Figure S10. I vs. t (min) curves for the accumulation of 20C charge at all the potentials from -0.75 V to -0.95V for ZrBTB@Hemin in the chronoamperometric measurement.

Table S2: Comparison of catalytic activity of molecular catalysts[(a)].

Catalyst	Electrolyte solution	% Faradaic Efficiency	TON	TOF (S^{-1})	Reference
Zr-BTB@Tandem	0.5M $KHCO_3$	15	514	0.43	This work
Mixture of CoPc and CNT	0.5M $KHCO_3$	0.3	44	~0.03[(b)]	2
CoPc/CNT	0.1M $KHCO_3$	44	3800	1.05	9
CoPc-NH_2/CNT	0.1M $KHCO_3$	32	3600	1.01	9
CoPc	0.5 M Na_2SO_4 with pH=3 acid solution	<5	N/A	~0.005[(a)]	10
Cu-ATSM	0.1 M KHCO3	74	N/A	0.79	11
$[(Phen)_2Ru(dppz)]^{2+}$	DMF with 0.30 mM $[(Phen)_2Ru(dppz)]^{2+}$ 0.1 M $TBAPF_6$ and 1 M H_2O	~0.2 (24 h)	0.92	$4x10^{-4}$[(b)]	12

(a) Only, molecular catalysts which reduces CO_2 to CH_3OH and where TOF value is reported, have been shown here.
(b)The value stated here has been reproduced from the table represented in ref. *8*

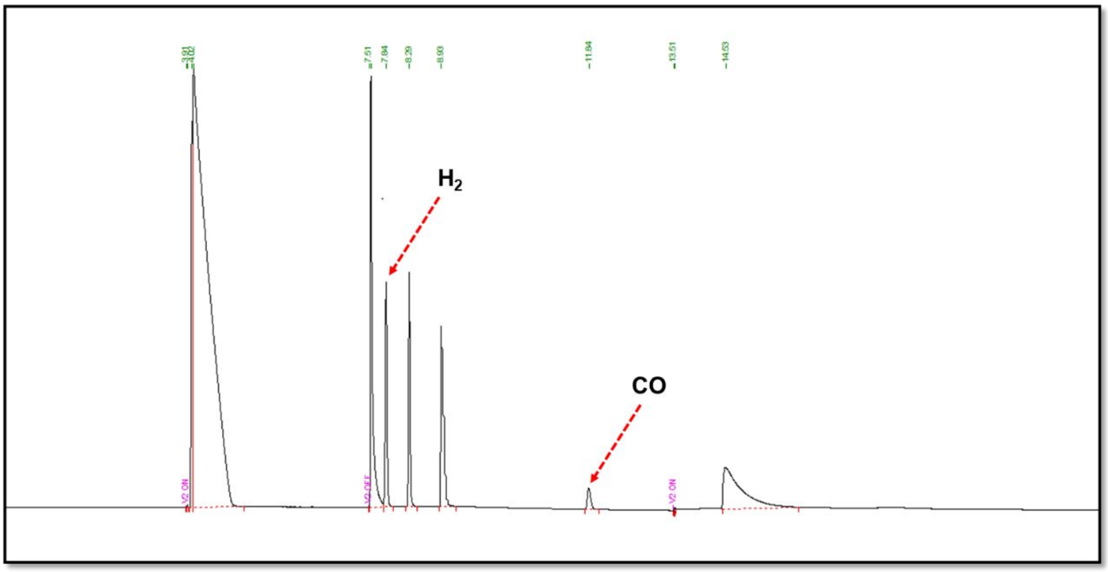

Figure S11. Representative GC profile, obtained for CO and H_2 quantification in case of all the three catalysts, from where FE of the minor product was calculated.

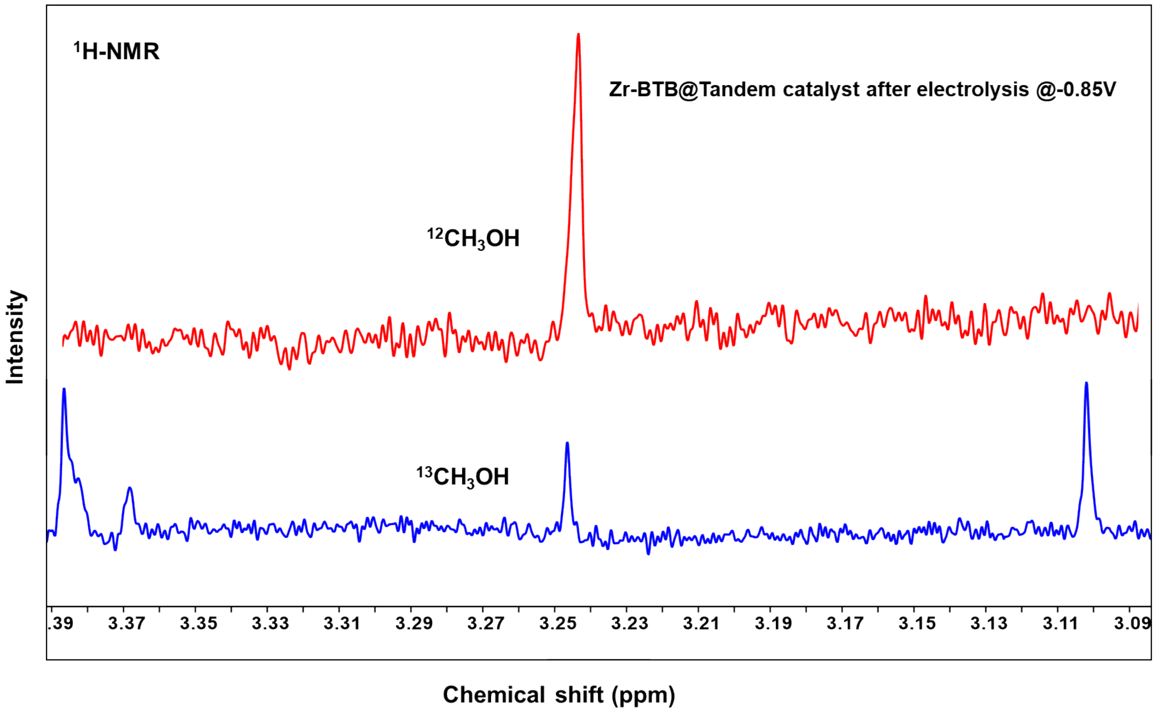

Figure S12. Representative ^{1}H NMR spectrum of $^{12}CH_3OH$ (red trace) and $^{13}CH_3OH$ (blue trace) obtained after electrolysis with $^{12}CO_2$ saturated and $^{13}CO_2$ saturated 0.5M $KHCO_3$ solution in presence of Zr-BTB@Tandem and Zr-BTB@CoPc catalysts at -0.85V vs. RHE.

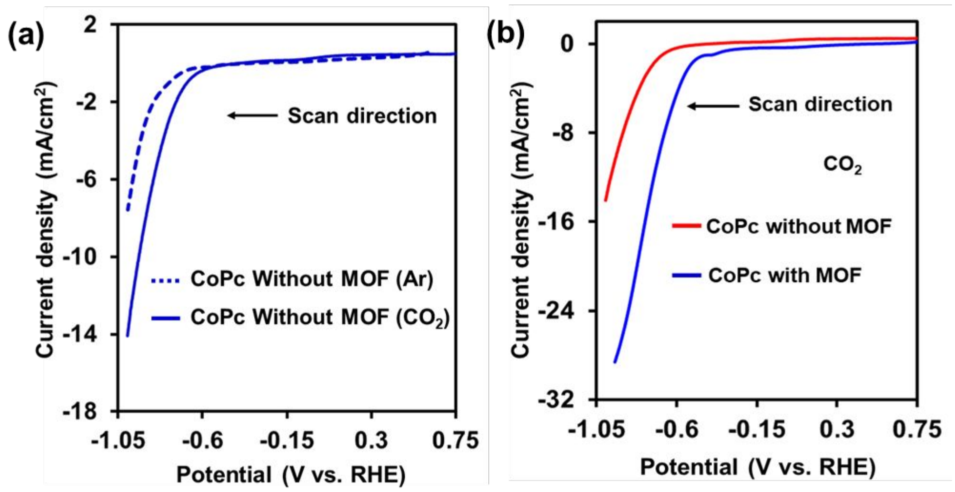

Figure S13. (a) Overlay of LSV data for CoPc without Zr-BTB MOF under Ar atmosphere (dotted trace) and under CO_2 atmosphere (solid trace), respectively. (b) Overlay of CO2RR catalytic activity of CoPc in absence of MOF (red trace) and in presence of MOF (blue trace), respectively.

The role of Zr-BTB MOF is to provide heterogeneous immobilization of a large concentration of molecular catalysts (CoPc and Hemin in the present system) to drive electrochemical catalysis, due to its highly modular nature and high surface area. Zr-BTB MOF has been used as a porous platform for the assembly of extremely high density of catalytically active sites (molecular catalysts) while providing mass-transport conduits accessible for diffusion of ions and catalytic-substrates towards the active sites. Apart from this in general, due to the well- defined nature and chemical modularity of MOFs to modulate the active site's chemical environment they do not require the catalytic and co-catalytic moieties to be covalently linked to achieve cooperative behaviour, unlike homogeneous molecular catalysts which require a great deal of synthetic precision to do so. That's why in our case we can rather easily implement the idea of tandem catalysis taking help from Zr-BTB. Therefore, the use of MOF (Zr-BTB, in this study) has several advantages over conventional dense heterogeneous solids.

We have loaded the same amount of CoPc catalyst without MOF over carbon cloth electrode, in order to test the activity in absence of Zr-BTB MOF. Figure S13a, shows the overlap of the LSV data of CoPc without MOF in presence of Ar (dotted trace) and in presence of CO_2 (solid trace), where it's clear that CoPc alone is also active towards CO_2RR, but the catalytic activity is much less compared to that in presence of MOF. Figure S13b shows the overlay of CO_2RR activity for CoPc, which clearly indicated less current density at high overpotential (with high Eonset) for CoPc without MOF (red trace) compared to that inside MOF (blue trace). That's why corresponding chronoamperometry at -0.85 V followed by ^{1}H NMR measurement indicates only 0.9% FE for CH_3OH formation, whereas in case of Zr-BTB@CoPc it was 5%. This is because, although we have same loading of catalysts in both the cases, yet the fraction of electroactive species in case of bare CoPc is much less due to aggregation as mentioned above.

Moreover, from the integration of the redox peak in the CV data of the Zr-BTB@Tandem catalyst system, we have obtained the surface concentration of electroactive species, 1.6 x 10^{-8} mol/cm^2. Knowing the total surface loading via ICP-OES, the electroactive catalyst fraction (in %) in the MOF-based tandem was calculated to be ~12%. Importantly, when higher electrolyte ionic strength was used (as in the case of our GDE experiments where 1M electrolyte was used, instead of only 0.1M electrolyte in H-cell), a larger fraction of molecular catalytic units were electro-activated (78%), due to the fact that MOF-based redox conduction (i.e self-exchange) is highly influenced by the concentration of supporting electrolyte counter ions.[13] On the contrary, we have conducted the CO_2RR experiments without the MOF platform, loading the same amount of CoPc catalysts over bare carbon cloth electrode. The fraction of electroactive species in that case was determined as only ~2.3%. This data of fairly low fraction of electroactive species for bare CoPc, indicates that inside the MOF, each catalyst is separated in space, while without it, the aggregation takes place making a very small portion of the catalyst to be active for electrocatalysis (thus explaining why only residual methanol (FE < 1%) was produced using bare CoPc).

Table S3. Ratio of Zr-BTB@Tandem Catalysts vs. %FE of CH3OH

Catalyst (Zr-BTB@Tandem)	Co/Zr_6	Fe/Zr_6	Co/Fe	%FE of CH_3OH at -0.85V vs. RHE
Ratio 1	0.90	0.78	1.15	15.0
Ratio 2	1.56	0.34	4.50	10.0
Ratio 3	0.75	0.92	0.82	5.40
Ratio 4	0.37	0.97	0.38	4.80
Ratio 5	0.65	0.59	1.10	12.0

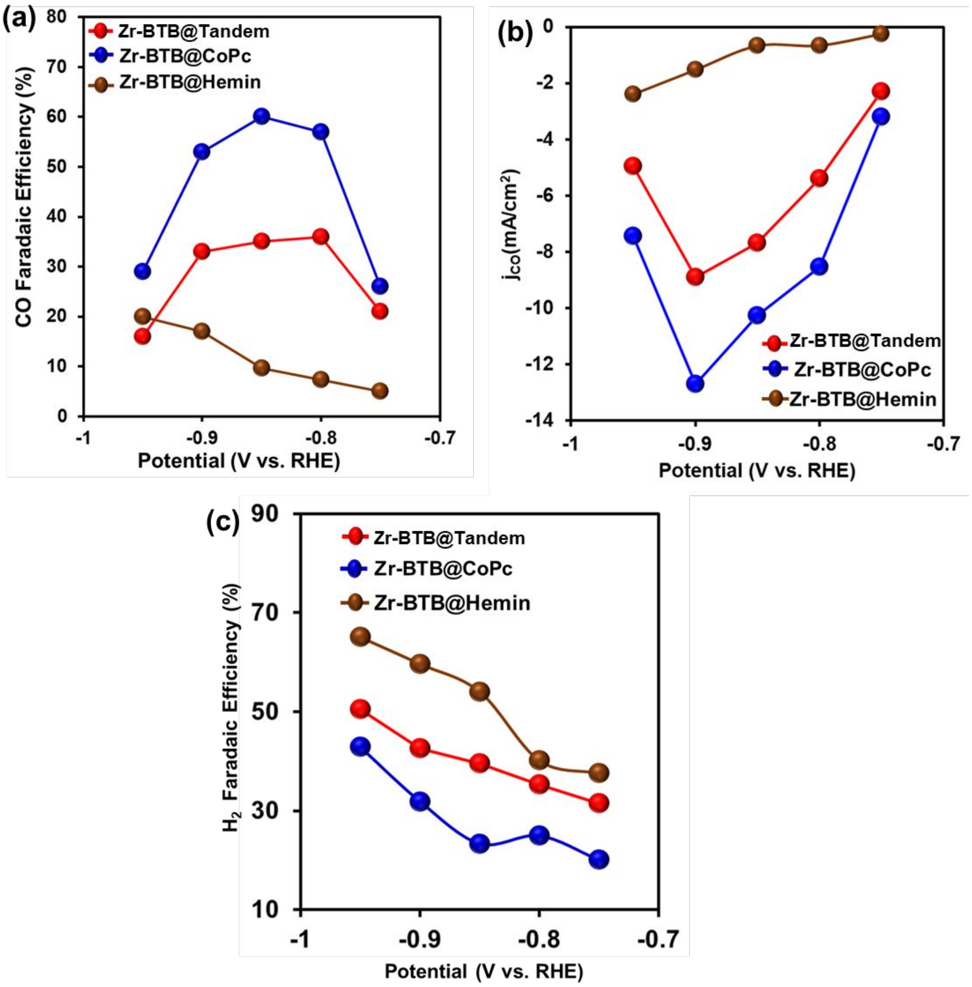

Figure S14. (a) Faradaic efficiency of CO in CO_2RR is plotted as a function of potential for Zr-BTB@Tandem (red trace) Zr-BTB@CoPc (blue trace) and Zr-BTB@Hemin (brown trace) in the potential range of -0.75 V to -0.95 V. (b) Partial current density (j_{CO}) vs. potential (V vs. RHE) for Zr-BTB@Tandem (red) and Zr-BTB@CoPc (blue) respectively in case of CO_2RR. (c) Faradaic efficiency of H_2 in CO_2RR is plotted as a function of potential for Zr-BTB@Tandem (red trace) Zr-BTB@CoPc (blue trace) and Zr-BTB@Hemin (brown trace) in the potential range of -0.75 V to -0.95 V.

Although there are various CO_2RR pathways, the competing hydrogen evolution reaction (HER) which has the similar reduction potential to that of CO_2RR, is the major reason behind the decreasing efficiency of CO_2RR conversion. Moreover, in case of molecular electrocatalysts the low valent electron-rich metal centers, which are deployed to activate CO_2 are prone to proton reduction as well, especially under aqueous electrolyte where the abundance of proton is huge (which is also a substrate for CO_2RR). Protonation of the reduced metal center produces a metal hydride (M-H) species, then depending upon its hydricity, the species can react with H^+ to produce H_2 or react with CO_2 to form HCOOH, where former is predominant as it is more spontaneous in aqueous medium. Alternatively, the challenge is to competitively bind CO_2 directly to the metal center via less Lewis acidic C atom, ruling out completely the metal hydride formation pathway and promoting CO_2RR to CO or other hydrocarbons.[14]In case of our catalyst systems also, these are the reasons why competing proton reduction jeopardizes the selectivity of the product from CO_2RR, especially where CoPc is not used. In case of Zr-BTB@Hemin, even within the presence of 2D-MOF also, the activation of Fe^{I} state is not

quite there for CO_2RR causing higher HER and hence more overpotential (energy) might have been required to go to the formal Fe^0 state to get more efficiency in CO_2 reduction.

In order to move forward, scientists all over the world are trying different strategies to suppress HER to promote CO_2RR. First of all, design of molecular catalysts can be done taking inspiration from natural enzymes, such as Ni-CODH type bioinspired ligand has been incorporated inside the molecular catalysts where thiolate ligand gets protonated keeping the metal center free for CO_2 binding. In this way, the metal center gets positive shift in reduction potential making the overpotential of overall CO_2RR process low.[15] Also, introducing second sphere, basic distal residues to the metal macrocycle, which gets protonated during the course of reaction can act as a proton transfer residue and promote CO_2RR over HER.[16] Apart from this, under aqueous heterogeneous condition control of mass transport condition is necessary for selective CO_2RR, where enhanced mass transport near the electrode may mitigate the increased HER rate and selectivity toward CO_2RR can be improved by increasing the thickness/roughness of the porous channels of the catalyst.[17] In this context, use of flow system can be very effective. Gas diffusion electrode (GDE) can solve the solubility and diffusivity limitation of CO_2 gas in liquid based electrocatalytic system, enhancing the selectivity of CO_2RR. Also, controlling the pH of the electrolyte medium, may help in suppressing HER as well.[18]

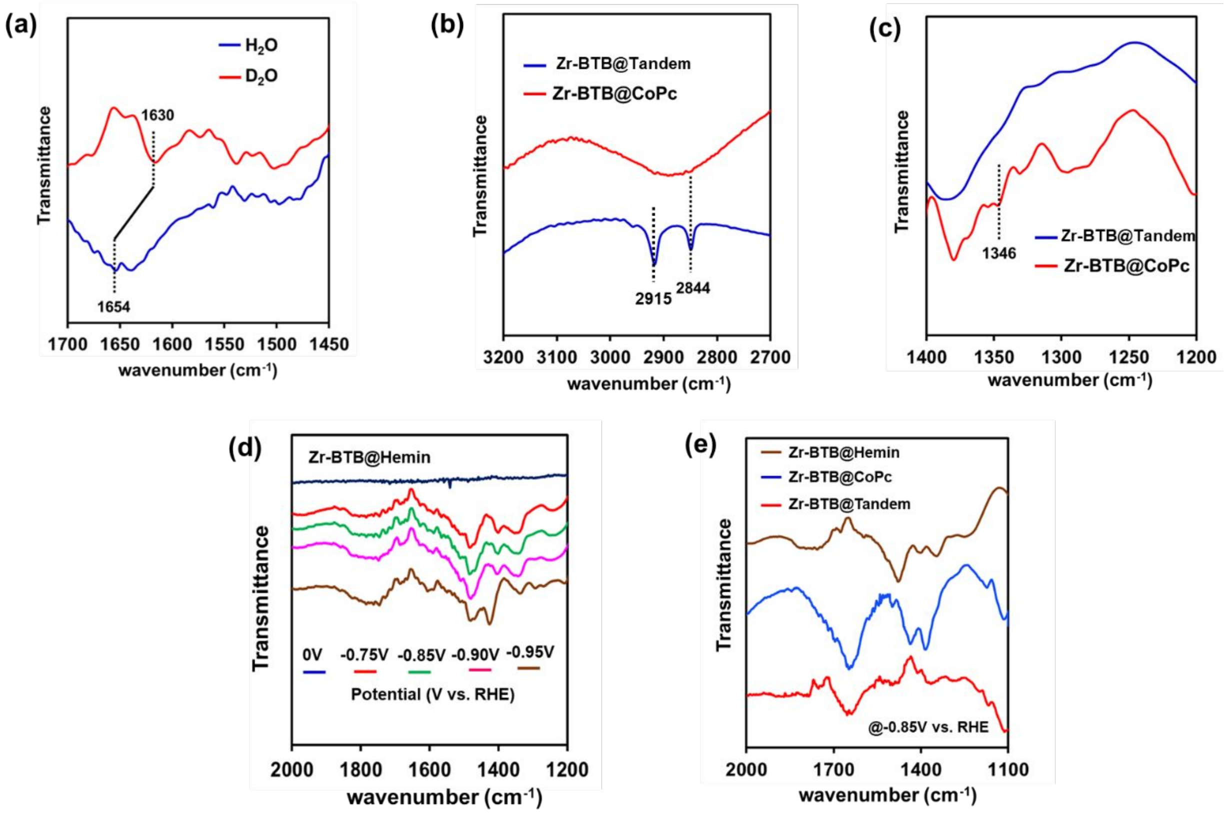

Figure S15. (a) Overlay of the ATR-IRRAS spectrum of Zr-BTB@Tandem catalyst showing the H (blue trace)/D(red trace) shift of the metal bound -CHO* intermediate species, obtained at -0.85V potential vs. RHE. (b) Comparison of the in-operando ATR-IRRAS data of Zr-BTB@Tandem (blue) and Zr-BTB@CoPc (red) in the high frequency region indicating stretching vibration ν(C-H) of metal bound methoxy ($-OCH_3$) species, present in Zr-BTB@Tandem only. (c) Comparison of the in-operando ATR-

IRRAS data of Zr-BTB@Tandem (blue) and Zr-BTB@CoPc (red) in the low frequency region indicating C-H bending of metal-bound OCH_3, i.e. δ(C-H), present in case of Zr-BTB@Tandem only. (d) In-operando ATR-IRRAS spectra of Zr-BTB@Hemin catalyst in $^{12}CO_2$ saturated 0.5 M aqueous $KHCO_3$ electrolyte starting from -0.75 V vs. RHE (red trace) to -0.95 V vs. RHE (brown trace). The spectrum taken at 0 V vs. RHE (blue trace) was considered as a background, where CO_2RR doesn't take place. (e) Overlay of the in-situ FTIR data of the three catalysts systems at -0.85V vs. RHE.

As can be seen in Figure S15d, in case of Zr-BTB @Hemin as the potential is gradually increased cathodically, there is no peak found in the ~ 1640 wavenumber region, indicating that for this system metal bound formyl species is not present. The overlay of the in-operando FTIR data at -0.85V vs. RHE for all the three catalysts (Figure S15e) also confirms that for Zr-BTB@Hemin the formyl species is not getting accumulated over the electrode during CO_2RR.

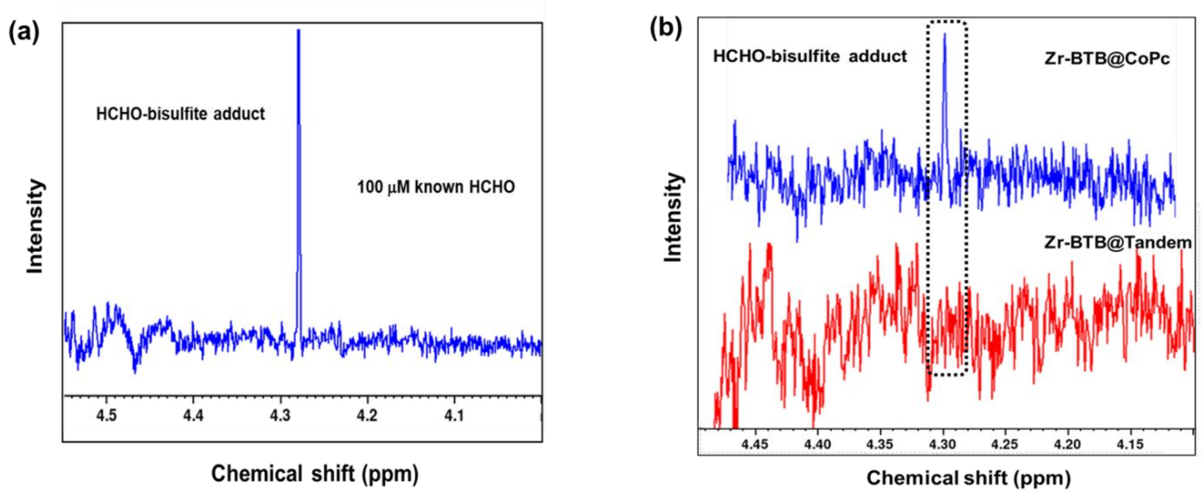

Figure S16. (a) Representative ^{1}H NMR spectrum of HCHO-bisulfite adduct recorded in D_2O solvent, obtained from the mixture of 100 µM HCHO solution and 1 M $NaHSO_3$ solution (50:50 v/v), from where the calibration curve was made. (b) ^{1}H NMR spectrum of HCHO-bisulfite adduct recorded in D_2O solvent for Zr-BTB@CoPc (blue) and Zr-BTB@Tandem catalyst (red), obtained after passing 500C charge at -0.95V vs. RHE in chronoamperometry experiment in each case.

Zr-BTB@CoPC showed a ^{1}H NMR (500 MHz) signal at 4.3 ppm for the HCHO-bisulfite adduct whereas in the case of Zr-BTB@Tandem catalyst the signal was absent. Although it is trace amount; ~ 0.2% FE of HCHO to be precise (as an intermediate from CO_2RR), yet this further proves the mechanism that we discussed in our system. It is actually the role of Hemin in the tandem system to facilitate the reaction from metal bound formyl state to CH_3OH formation.

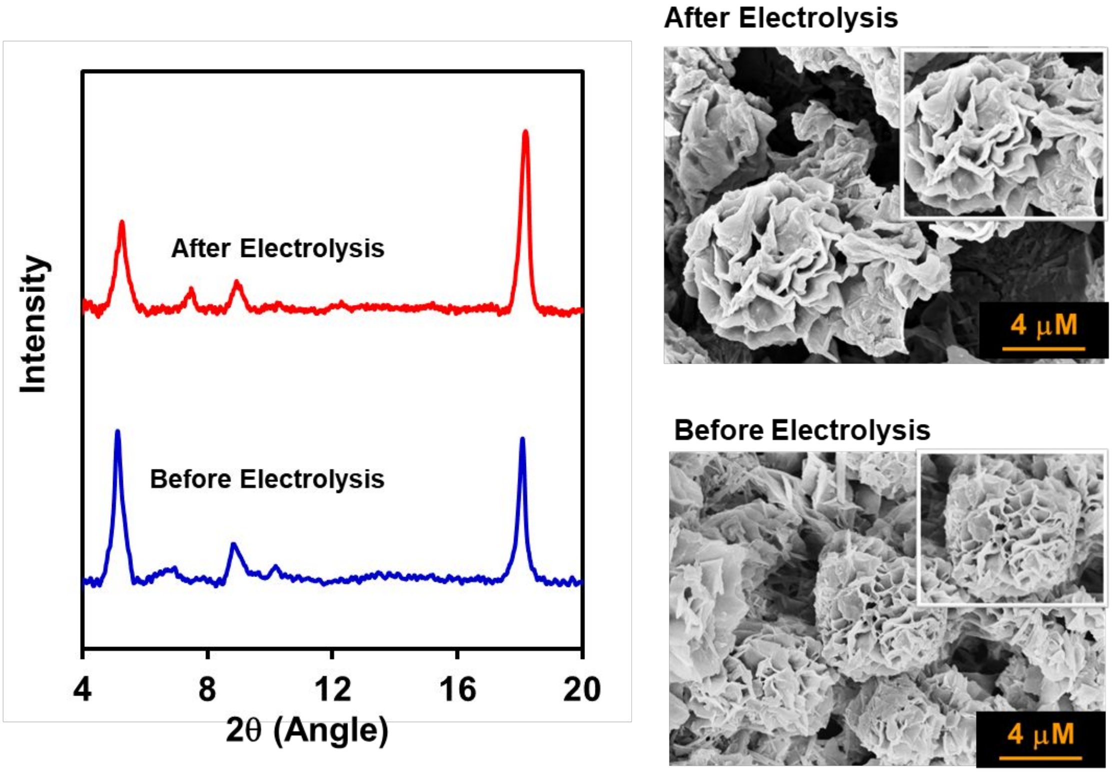

Figure S17. (a) PXRD pattern of the Zr-BTB@Tandem catalyst from GDE before electrolysis (blue) and after 3 hrs. of electrolysis (red trace). (b) SEM images of the Zr-BTB@Tandem catalyst from GDE electrode before electrolysis and after 4 hours of electrolysis, showing minimal changes in morphology.

Note: We delve deeper into the comparative study of our system's efficacy in flow cell. McCrory and co-workers demonstrated in GDE- flow cell[19] competitive CO_2 binding to CoPc rather than CO takes place even at moderate CO_2 local concentration and hence by using only single pass electrolyte this issue cannot be fully solved, since the inherent affinity of CO_2 binding to CoPc is more compared to CO. In that aspect, our tandem system which proceeds via formyl bound intermediate may add flexibility. McCory and co-workers have mentioned construction of catalyst−polymer composites, or via applying modifications to the coordination environments of CoPc as probable remedies. As demonstrated by Robert and co-workers in a recirculating flow cell they get CO_2 to CO with 95 % FE and by using pure CO as pure reactant in a sequential manner got 14.3% CH_3OH and that too at pH 13, by using CoPc catalyst.[2] While Lei Wang and co-workers showed in a recirculating flow cell in 1M $KHCO_3$ 10.5% CH_3OH selectivity from CO_2RR, although with high partial current density of 84 mA cm^{-2}.[20] Not at physiological pH but in presence of mixture of 0.2M KOH and 1.5M KCl solution Ye and co-workers were able to produce 38.5% FE of methanol with high partial current density from CO2RR with the catalyst CoTMAPc in recirculating flow cell (modified phthalocyanine).[21]

Therefore, our tandem-based catalyst system is very effective in-terms of methanol catalytic activity from direct CO_2RR if we compare other data from recirculating flow cell, especially under physiological pH.

**Table S4.** Data sheet obtaining the average value reported in manuscript with error bar				
**Catalyst**	**Potential (V vs. RHE)**	**Avg. %FE with standard deviation**	**Avg. total current passed with standard deviation (mA/cm^2)**	**Avg. partial current density (mA/cm^2)**
Zr-BTB@Tandem for CO_2RR to CH_3OH in H-cell	-0.75	5.08 ± 0.38	-11.40 ± 0.05	-0.58
	-0.80	10.90 ± 0.50	-15.00 ± 1.50	-1.64
	-0.85	15.00 ± 1.40	-22.00 ± 2.80	-3.30
	-0.90	13.40 ± 0.80	-27.00 ± 3.00	-3.62
	-0.95	8.09 ± 1.02	-31.25 ± 2.00	-2.53
Zr-BTB@Tandem for HCHO reduction to CH_3OH in H-cell	-0.85	18.00 ± 1.52	-16.00 ± 0.84	-2.88
	0.90	12.50 ± 0.65	-24.48 ± 3.10	-3.06
	-0.95	14.00 ± 0.66	-32.00 ± 3.68	-4.48
Zr-BTB@Tandem for CO_2RR to CH_3OH in GDE	-1.0 (30 min)	18.00 ± 0.17	-138.00 ± 2.00	-24.84
	-1.0 (60 min)	15.90 ± 0.98	-150.40 ± 4.20	-23.91
	-1.0 (120 min)	14.20 ± 0.30	-176.50 ± 1.55	-25.06
	-1.0 (240 min)	13.80 ± 0.33	-184.60 ± 2.11	-25.39
Zr-BTB@CoPc for CO_2RR to CH_3OH in H-cell	-0.75	1.00 ± 0.20	-12.10 ± 0.04	-0.12
	-0.80	3.50 ± 0.90	-14.50 ± 0.65	-0.51
	-0.85	5.00 ± 0.61	-17.20 ± 1.51	-0.86
	-0.90	4.10 ± 1.30	-24.00 ± 0.60	-0.98
	-0.95	4.60 ± 0.48	-25.70 ± 1.85	-1.02
Zr-BTB@CoPc for HCHO reduction to CH_3OH in H-cell	-0.85	10.43 ± 0.99	-16.30 ± 1.20	-1.70
	0.90	8.74 ± 0.60	-19.00 ± 1.00	-1.66
	-0.95	9.50 ± 0.95	-23.70 ± 2.25	-2.25
Zr-BTB@Hemin for HCHO reduction to CH_3OH in H-cell	-0.85	25.0 ± 1.50	-10.40 ± 0.74	-2.60
	0.90	15.06 ± 0.72	-13.80 ± 2.01	-2.08
	-0.95	11.58 ± 0.60	-28.50 ± 2.67	-3.30

Reference

(1) Shimoni, R.; Shi, Z.; Binyamin, S.; Yang, Y.; Liberman, I.; Ifraemov, R.; Mukhopadhyay, S.; Zhang, L.; Hod, I. Electrostatic Secondary-Sphere Interactions That Facilitate Rapid and Selective Electrocatalytic CO_2 Reduction in a Fe-Porphyrin-Based Metal–Organic Framework. *Angew. Chem. Int. Ed.* **2022**, *61* (32), e202206085.

(2) Boutin, E.; Wang, M.; Lin, J. C.; Mesnage, M.; Mendoza, D.; Lassalle-Kaiser, B.; Hahn, C.; Jaramillo, T. F.; Robert, M. Aqueous Electrochemical Reduction of Carbon Dioxide and Carbon Monoxide into Methanol with Cobalt Phthalocyanine. *Angew. Chem. Int. Ed.* **2019**, *58* (45), 16172-16176.

(3) Chatterjee, T.; Boutin, E.; Robert, M. Manifesto for the Routine Use of NMR for the Liquid Product Analysis of Aqueous CO_2 Reduction: from Comprehensive Chemical Shift Data to Formaldehyde Quantification in Water. *Dalton Trans.* **2020**, *49* (14), 4257-4265.

(4) Ghatak, A.; Shanker, G. S.; Sappati, S.; Liberman, I.; Shimoni, R.; Hod, I. Pendant Proton-Relays Systematically Tune the Rate and Selectivity of Electrocatalytic Ammonia Generation in a Fe-Porphyrin Based Metal–Organic Framework. *Angew. Chem. Int. Ed.* **2024**, *63* (37), e202407667.

(5) Mukhopadhyay, S.; Naeem, M. S.; Shiva Shanker, G.; Ghatak, A.; Kottaichamy, A. R.; Shimoni, R.; Avram, L.; Liberman, I.; Balilty, R.; Ifraemov, R.; et al. Local CO_2 Reservoir Layer Promotes Rapid and Selective Electrochemical CO_2 Reduction. *Nat. Commun.* **2024**, *15* (1), 3397.

(6) Srinet, G.; Varshney, P.; Kumar, R.; Sajal, V.; Kulriya, P. K.; Knobel, M.; Sharma, S. K. Structural, Optical and Magnetic Properties of $Zn_{1-x}Co_xO$ Prepared by the Sol–Gel Route. *Ceram. Int.* **2013**, *39* (6), 6077-6085.

(7) Batchelor-McAuley, C. Defining the Onset Potential. *Curr. Opin. Electrochem.* **2023**, *37*, 101176.

(8) Blasco-Ahicart, M.; Soriano-López, J.; Carbó, J. J.; Poblet, J. M.; Galan-Mascaros, J. R. Polyoxometalate Electrocatalysts Based on Earth-Abundant Metals for Efficient Water Oxidation in Acidic Media. *Nat. Chem.* **2018**, *10* (1), 24-30.

(9) Wu, Y.; Jiang, Z.; Lu, X.; Liang, Y.; Wang, H. Domino Electroreduction of CO_2 to Methanol on a Molecular Catalyst. *Nature* **2019**, *575* (7784), 639-642.

(10) Kapusta, S.; Hackerman, N. Carbon Dioxide Reduction at a Metal Phthalocyanine Catalyzed Carbon Electrode. *J. Electrochem. Soc.* **1984**, *131* (7), 1511-1514.

(11) Jiang, W.-X.; Liu, W.-X.; Wang, C.-L.; Zhan, S.-Z.; Wu, S.-P. A Bis(thiosemicarbazonato)-Copper Complex, a New Catalyst for Electro- and Photo-Reduction of CO_2 to Methanol. *NJC* **2020**, *44* (7), 2721-2726.

(12) Boston, D. J.; Pachón, Y. M. F.; Lezna, R. O.; de Tacconi, N. R.; MacDonnell, F. M. Electrocatalytic and Photocatalytic Conversion of CO_2 to Methanol using Ruthenium Complexes with Internal Pyridyl Cocatalysts. *Inorg. Chem.* **2014**, *53* (13), 6544-6553.

(13) Kung, C.-W.; Goswami, S.; Hod, I.; Wang, T. C.; Duan, J.; Farha, O. K.; Hupp, J. T. Charge Transport in Zirconium-Based Metal-Organic Frameworks. *Acc. Chem. Res.* **2020**, *53* (6), 1187-1195.

(14) Saha, P.; Amanullah, S.; Dey, A. Selectivity in Electrochemical CO_2 Reduction. *Acc. Chem. Res.* **2022**, *55* (2), 134-144.

(15) Dey, S.; Ahmed, M. E.; Dey, A. Activation of Co(I) State in a Cobalt-Dithiolato Catalyst for Selective and Efficient CO_2 Reduction to CO. *Inorg. Chem.* **2018**, *57* (10), 5939-5947.

(16) Amanullah, S.; Saha, P.; Dey, A. Activating the Fe(I) State of Iron Porphyrinoid with Second-Sphere Proton Transfer Residues for Selective Reduction of CO_2 to HCOOH via Fe(III/II)–COOH Intermediate(s). *J. Am. Chem. Soc.* **2021**, *143* (34), 13579-13592.

(17) Goyal, A.; Marcandalli, G.; Mints, V. A.; Koper, M. T. M. Competition between CO_2 Reduction and Hydrogen Evolution on a Gold Electrode under Well-Defined Mass Transport Conditions. *J. Am. Chem. Soc.***2020**, *142* (9), 4154-4161.

(18) Khalil, M.; Kadja, G. T. M.; Nugroho, F. A. A.; Sutanto, L. G.; Jiwanti, P. K.; Abdi, F. F.; Hussin, F.; Aroua, M. K. Suppressing the Competing Hydrogen Evolution Reaction in CO_2 Electroreduction: A Review. *Renew. Sustain. Energy Rev.* **2024**, *206*, 114869.

(19) Yao, L.; Rivera-Cruz, K. E.; Zimmerman, P. M.; Singh, N.; McCrory, C. C. L. Electrochemical CO_2 Reduction to Methanol by Cobalt Phthalocyanine: Quantifying CO_2 and CO Binding Strengths and Their Influence on Methanol Production. *ACS Catal.* **2024**, *14* (1), 366-372.

(20) Yang, H.; Guo, N.; Xi, S.; Wu, Y.; Yao, B.; He, Q.; Zhang, C.; Wang, L. Potential-Driven Structural Distortion in Cobalt Phthalocyanine for Electrocatalytic CO_2/CO Reduction towards Methanol. *Nature Communications* **2024**, *15* (1), 7703.

(21) Song, Y.; Guo, P.; Ma, T.; Su, J.; Huang, L.; Guo, W.; Liu, Y.; Li, G.; Xin, Y.; Zhang, Q.; et al. Ultrathin, Cationic Covalent Organic Nanosheets for Enhanced CO_2 Electroreduction to Methanol. *Adv. Mater.* **2024**, *36* (17), 2310037.
